# Nanotechnology to Overcome Blood–Brain Barrier Permeability and Damage in Neurodegenerative Diseases

**DOI:** 10.3390/pharmaceutics17030281

**Published:** 2025-02-20

**Authors:** Adriana Jiménez, Enrique Estudillo, Mara A. Guzmán-Ruiz, Nieves Herrera-Mundo, Georgina Victoria-Acosta, Enoc Mariano Cortés-Malagón, Adolfo López-Ornelas

**Affiliations:** 1 División de Investigación, Hospital Juárez de México, Ciudad de México 07760, Mexico; adriana.jimenez@salud.gob.mx (A.J.); georgina.acostaa@salud.gob.mx (G.V.-A.); emcortes@cinvestav.mx (E.M.C.-M.); 2 Laboratorio de Reprogramación Celular, Instituto Nacional de Neurología y Neurocirugía Manuel Velasco Suárez, Ciudad de México 14269, Mexico; jestudillo@innn.edu.mx; 3 Departamento de Fisiología, Facultad de Medicina, Universidad Nacional Autónoma de México, Ciudad de México 04510, Mexico; mara_alaide@comunidad.unam.mx; 4 Departamento de Biología Celular y Fisiología, Instituto de Investigaciones Biomédicas, Universidad Nacional Autónoma de México, Ciudad de México 04510, Mexico; nieves_herrera@iibiomedicas.unam.mx; 5 Hospital Nacional Homeopático, Hospitales Federales de Referencia, Ciudad de México 06800, Mexico

**Keywords:** nanotechnology, nanoparticles, neurodegenerative diseases, blood–brain barrier, stem cells

## Abstract

The blood–brain barrier (BBB) is a critical structure that maintains brain homeostasis by selectively regulating nutrient influx and waste efflux. Not surprisingly, it is often compromised in neurodegenerative diseases. In addition to its involvement in these pathologies, the BBB also represents a significant challenge for drug delivery into the central nervous system. Nanoparticles (NPs) have been widely explored as drug carriers capable of overcoming this barrier and effectively transporting therapies to the brain. However, their potential to directly address and ameliorate BBB dysfunction has received limited attention. In this review, we examine how NPs enhance drug delivery across the BBB to treat neurodegenerative diseases and explore emerging strategies to restore the integrity of this vital structure.

## 1. Introduction

Neurodegenerative diseases, including Alzheimer’s, Parkinson’s, Huntington’s, and amyotrophic lateral sclerosis (ALS), represent a significant global health burden, affecting millions of individuals and imposing immense socioeconomic costs [1]. They lead to direct expenses, such as hospital stays, medication, and long-term care, as well as indirect costs, such as informal caregiving, productivity loss, and increased demands on social services. For example, in 2020, dementia was estimated to affect 50 million people worldwide, incurring over USD 1 trillion in global expenses as of 2018, a figure that may exceed USD 2 trillion by 2030 [2,3,4]. These trends strain healthcare infrastructures and place heavy emotional and financial burdens on caregivers [5]. In light of these challenges, robust public health policies focusing on early detection, prevention, caregiver support, and continued research, particularly cutting-edge treatments such as nanotechnology and regenerative medicine, remain pivotal for mitigating their broad impact [6].

According to the World Health Organization, Alzheimer’s disease (AD), the most common neurodegenerative disorder, affects approximately 55 million people worldwide as of 2021, a figure that is projected to double by 2050 [1]. Parkinson’s disease (PD) affects over 8.5 million individuals globally, with the prevalence increasing with age, particularly in those over 60 years of age [7]. ALS, though less prevalent, affects two to five people per 100,000 each year, placing a substantial burden on quality of life and healthcare systems [8].

Neurodegenerative disorders such as AD are characterized by the accumulation of amyloid-beta (Aβ) plaques, tau tangles, and neuronal death, ultimately leading to cognitive decline [9]. Similarly, PD involves the progressive loss of dopaminergic neurons, resulting in debilitating motor symptoms [10]. ALS is characterized by the degeneration of motor neurons, leading to severe muscle atrophy and eventually respiratory failure [11].

The blood–brain barrier (BBB) is a selective structure that preserves the homeostasis of the central nervous system (CNS) by regulating nutrient transport, facilitating waste removal, and restricting the entry of harmful agents as well as the trafficking of peripheral immune cells to the CNS. The BBB consists of a layer of brain endothelial cells (BECs) connected by tight junctions (TJs), forming an interface between the blood and the brain. BECs are surrounded by a basement membrane, pericytes, and astrocytic endfeet that interact with neurons and microglia to regulate BBB permeability and blood flow. These components, collectively known as the neurovascular unit (NVU), maintain BBB integrity and function [12,13].

The integrity of the BBB decreases with age, but it is also strongly affected in neurodegenerative diseases. Under these conditions, pathological processes such as neuro-inflammation and oxidative stress can cause damage to BECs and pericytes, a reduction in TJ proteins, and changes in transport across the BBB [14]. The role of the BBB in neurodegenerative disorders highlights the importance of developing methods for its restoration. However, although this structure is altered in neurodegenerative diseases, it remains an obstacle that restricts the entry of many therapeutic drugs into the brain. Therefore, this review aims to describe potential strategies involving nanoparticles (NPs) to restore both BBB integrity and function and overcome BBB permeability.

## 2. Blood–Brain Barrier Dysfunction in Neurodegenerative Diseases

Several investigations have associated BBB damage with neurodegenerative and neurological diseases. In AD, a compromised BBB is observed in humans and animal models of the disease. Increased BBB permeability in AD has been demonstrated by the presence of proteins such as albumin in the prefrontal cortex, entorhinal cortex, and hippocampus [15]. AD is characterized by the accumulation of senile plaques formed by the deposition of Aβ peptides and neurofibrillary tangles (NFTs) produced by the phosphorylation and aggregation of the tau protein. BBB damage in AD may be partially caused by the aggregation of Aβ and tau, as senile plaques and NFTs are associated with the thickening of the cerebral microvasculature [16]. Vascular deposition of Aβ has been shown to promote immune cell infiltration into the CNS by decreasing the levels of TJ proteins in BECs and increasing BBB permeability, thereby promoting neuro-inflammation [17]. Moreover, disruption of TJs results from pericyte degeneration and uncoupling of BECs and astrocytes, leading to increased leakage of proteins such as IgG and fibrin across the BBB [18].

Postmortem studies of the brains of AD patients have demonstrated an increased accumulation of blood-derived proteins due to BBB permeability [19]. This phenomenon has been associated with a reduction in TJ protein expression [20] and reduced P-glycoprotein (P-gp) function [21]. In addition, patients with AD present reductions in the uptake of [(18)F] fluorodeoxyglucose (FDG) in the temporoparietal regions and posterior cingulate gyrus [22], which has been associated with a reduction in glut 1 and 3 transporters [23]. On the other hand, experiments in a rodent model of AD demonstrated that BECs have a decrease in the Wnt pathway, and its restoration prevents BEC impairment, as Wnt signaling increases the expression of TJ-related genes such as *claudin-5* and *ZO-1*; likewise, Wnt signaling also restores BEC impermeability [24]. This information suggests that the disruption of the BBB in AD could be partially mediated by the impairment of Wnt signaling. Finally, ROS activity and an increase in inflammatory mediators have also been associated with alterations in the physiology of the different cell types that constitute the NVU and promote the migration of immune cells into cerebral vessels, further contributing to the pro-inflammatory state observed during neurodegeneration [25].

Changes in the molecular composition of the basement membrane can be observed in acute and chronic pathological conditions such as stroke and AD. The basement membrane is formed by extracellular matrix proteins synthesized and secreted by the cells of the NVU that provide support to endothelial cells, contributing to the integrity of the BBB. During stroke, the upregulation of proteases such as cathepsins B and L, which degrade perlecan, and the metalloproteases (MMPs) MMP-9 and MMP-2, which are involved in the degradation of fibronectin, collagen IV, and laminin, has been observed. Three main changes in the basement membrane have been described in AD: (1) Aβ deposition in the walls of the cerebral vasculature (known as cerebral amyloid angiopathy), which occurs in approximately 80% of AD cases; (2) basement membrane thickening in capillaries of AD patients and AD animal models; and (3) altered distribution of basal membrane proteins such as collagen IV, fibronectin, and perlecan; however, the changes in these proteins are inconsistent [26].

MMPs also promote the degradation of TJs, leading to the detachment of BECs and astrocytic endfeet, as well as the loss of pericytes [14]. MMP activation may occur via the inflammatory response triggered by microglia after CNS damage [27]. Therefore, BBB damage caused by MMPs is associated mainly with massive inflammatory processes, such as those that occur during a stroke. Disruption of blood flow in ischemic stroke induces necrosis, oxidative stress, leukocyte infiltration, and BBB damage. During stroke, BECs secrete MPP-2 and MPP-9, which participate in the degradation of TJs and the basement membrane [18,28]. On the other hand, the role of MMPs in chronic neurodegeneration has not been elucidated, but postmortem analysis of the cerebral cortex of AD patients and controls without dementia demonstrated that tissue from AD patients carrying the APOE4 isoform had increased permeability to fibrin and immunoglobulin G, which correlated with the degeneration of pericytes and the accumulation of the pro-inflammatory cytokines cyclophilin A and MMP-9 [29].

Alterations in the BBB have also been reported in PD. Cerebrospinal fluid examination demonstrated the presence of albumin infiltration in nondemented PD patients with advanced disease [30]. PD is characterized by the presence of Lewy bodies formed by the aggregation and deposition of α-synuclein. Lewy bodies accumulate and spread in different areas of the brain, but the characteristic motor symptoms of this disease are associated with the involvement of the nigrostriatal pathway and the death of dopaminergic neurons in the substantia nigra pars compacta [31]. Interestingly, histological analysis of brain samples from PD patients revealed the presence of serum proteins and iron and erythrocyte extravasation in the striatum, suggesting that BBB leakage in this brain region is related to PD pathology [32]. Furthermore, plasma protein leakage from subthalamic nucleus (STN) capillaries into the brain parenchyma was observed in postmortem PD samples; the STN projects to the internal segment of the globus pallidus and substantia nigra. STN microvessel leakage correlated with decreased immunoreactivity of the TJ proteins ZO-1, occludin, and claudin-5 and the cell adhesion protein VE-cadherin, whose expression levels were increased in samples from PD patients treated with STN deep brain stimulation [33].

In addition, oligomeric α-synuclein can impair BBB integrity through vascular endothelial growth factor (VEGF)-A secretion, since astrocyte stimulation with this oligomer triggers the release of VEGFA. Interestingly, VEGFA contributes to an impairment of the barrier properties of BECs in vitro, and further analyses in a rodent model of PD in mice revealed greater expression of VEGFA in astrocytes than in controls. Notably, postmortem samples from PD patients also presented elevated levels of VEGFA in astrocytes, suggesting that VEGFA derived from astrocytes contributes to BBB leakage in PD [34].

Transport across the BBB is affected in neurodegenerative disorders. The expression of the receptor for advanced glycation end products (RAGE) is increased in the brain endothelium of AD patients, whereas the expression of low-density lipoprotein receptor-related protein 1 (LRP1) is reduced [35]. Importantly, RAGE and LRP1 play key roles in the clearance of Aβ from the brain parenchyma, where RAGE facilitates its uptake from the blood circulation to the CNS, and LRP1 participates in its efflux [12]. Therefore, the altered expression of these proteins in BECs may influence the accumulation of Aβ in AD and disease progression.

Transcellular transport through BECs is also affected in PD. For example, it is hypothesized that reduced P-glycoprotein (P-gp) activity increases the risk of PD through the accumulation of neurotoxic substances, which is supported by the high level of verapamil (a P-gp substrate) observed in the midbrains of patients with PD [21]. Furthermore, P-gp plays a key role in Aβ clearance from the brain and is decreased in patients with AD and cerebral amyloid angiopathy [36]. In addition, hypometabolism in several neurodegenerative diseases has been attributed, in part, to decreased expression of glucose transporters in the BBB and neurons [37].

Neuro-inflammation is an important contributor to the neuronal damage observed in AD and PD. Aβ plaques promote the activation of microglia, which in turn produce pro-inflammatory factors such as tumor necrosis factor-α (TNF-α), interleukin-1β, interleukin-6 (IL-1β, IL-6), transforming growth factor-β (TGF-β), and reactive oxygen species (ROS). Moreover, BBB disruption in multiple sclerosis is considered essential for the infiltration of T cells and their interaction with microglia to induce neuro-inflammation [38]. In turn, TNF-α and IL-1β modulate BBB permeability by decreasing the expression of the TJ proteins ZO-1, occludin, claudin-3, and LRP1, thus increasing BBB permeability and decreasing Aβ efflux [39].

**Figure 1:**
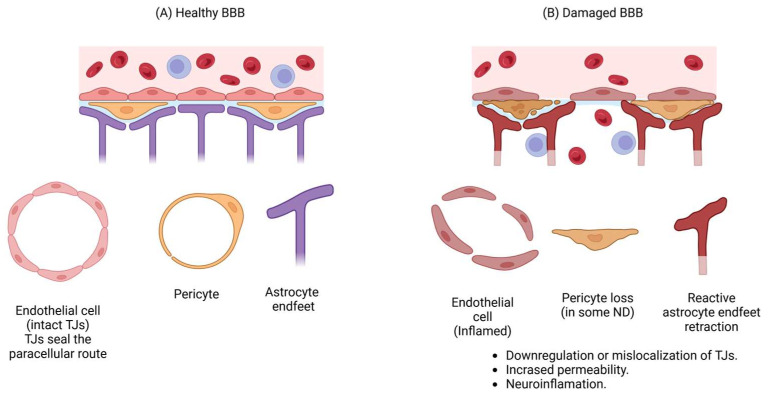
Schematic depiction of blood–brain barrier (BBB) integrity and dysfunction in neurodegenerative diseases. (**A**) Under healthy conditions, tight junction proteins (e.g., claudin-5, occludin, and ZO-1) form a highly selective seal between endothelial cells, with pericytes and astrocytic endfeet contributing to BBB stability and proper nutrient exchange. (**B**) In neurodegenerative pathologies (e.g., AD or PD), inflammatory processes, pericyte loss, and reduced tight junction expression lead to increased permeability, facilitating the infiltration of immune cells and toxic molecules into the brain parenchyma. Created in https://BioRender.com (accessed on 17 February 2025).

As mentioned previously, several mechanisms associated with neurodegeneration involve damage to the BBB; therefore, more research should be conducted to focus on the recovery of the BBB as a therapeutic target for neurodegenerative disorders (Figure 1).

Although the BBB is now considered to be a structure involved in different neurodegenerative and neurological diseases, several contrasting reports suggest that the BBB remains unaltered in neurodegenerative processes. For example, no BBB transport abnormalities were found in 14 individuals with probable AD compared with elderly control subjects [40]. An evaluation of BBB permeability in five AD patients and five age-matched controls via the tracer [68Ga]-ethylenediaminetetraacetic acid revealed no evidence of infiltration into the brain parenchyma [41]. Similar results were observed in 15 AD participants and 15 healthy older subjects administered Gd-DTPA, in which BBB leakage did not differ between the groups; however, different blood–brain–cerebrospinal fluid (CSF) compartmental kinetics were observed [42]. Furthermore, reduced uptake of IgG, anti-BACE1, and anti-TfR/BACE1 antibodies was observed in the brains of PS2-APP, P301L, and P301S AD model mice, in contrast to the high CNS uptake of these molecules in the experimental model of allergic encephalomyelitis, which causes massive damage to the BBB, suggesting that no or minimal BBB impairment occurs in AD [43].

## 3. Overview of Nanoparticle Transport Across the Blood–Brain Barrier

The BBB acts as a protective barrier that selectively restricts the entry of numerous substances into the brain, which is why several pharmacological strategies have been developed for the treatment of neurological and neurodegenerative diseases, such as brain tumors, epilepsy, AD, and PD, to increase the entry of drugs into the CNS. The pharmacotherapy available often faces the impossibility of effectively crossing the BBB to address the underlying causes of these diseases [44]. Even though some small molecules, such as acetylcholinesterase inhibitors and memantine, which are approved for the treatment of AD, can cross the BBB and enter the brain parenchyma, >98% of small molecules do not cross the BBB [45]; therefore, their biological activity is suboptimal [46,47,48], presenting a challenge for the development of CNS therapies [49].

NP-based systems have revolutionized drug delivery by incorporating therapeutic molecules into carriers that protect them from enzymatic degradation, prolong their circulation half-life, and allow for targeted release [50]. The BBB, however, is designed to limit the entry of both macromolecules and most small molecules into the CNS [51]. To circumvent this blockade, NP designs frequently exploit solute carrier (SLC) proteins responsible for transporting essential nutrients (e.g., glucose and amino acids) into the brain. By attaching ligands resembling these natural substrates onto the NP surface, researchers can “trick” receptor-mediated pathways into importing NP–drug complexes across the endothelial cell layer [52,53,54]. For example, transferrin-conjugated lipid–polymer hybrid NPs loaded with N-acetylcysteine (NAC) crossed the BBB in an in vitro AD model. These compounds significantly reduce inflammatory gene expression and cytokine secretion in human induced pluripotent stem cell (iPSC)-derived astrocytes. Compared with free NAC, these NPs showed enhanced anti-inflammatory efficacy, suggesting a promising approach to addressing neuro-inflammation in AD [55].

Careful substrate selection is essential for success. The targeted SLC must exhibit robust expression along the BBB, and substrate affinity needs to be well calibrated to ensure the release of the cargo in the brain rather than its entrapment in intracellular compartments such as lysosomes [56]. Moreover, inorganic NPs often cause concerns about instability and cytotoxicity [57], prompting scientists to develop strategies such as coating NPs with biocompatible materials, such as polyethylene glycol (PEG), poly(lactic-co-glycolic acid) (PLGA), chitosan, or polysorbate 80, to stabilize them and reduce adverse effects [58,59,60,61,62]. Additional structural properties (e.g., size, shape, stiffness, and overall composition) also prove critical for circulation time and intracellular trafficking; however, the functionalization of inorganic NPs is an efficient way to overcome some structural properties to facilitate their passage across BECs, since the functionalization of gold NPs with a monovalent TfR-targeting antibody (anti-TfR/BACE 1) increases their brain accumulation [63,64,65].

While the efficiency of NPs needs to be optimized to cross the BBB, different NP systems have shown efficacy for the treatment of CNS diseases; however, importantly, safety concerns, including the fate of NPs in the brain, remain unclear. NPs have less toxicity than free drugs do, but this depends on the characteristics of the NPs, such as the nanomaterial used, size, charge, coating, distribution, and dose. Studies have reported the toxic effects of gold and silica NPs, micelles, and poly(amidoamine) (PAMAM) dendrimers. Furthermore, bioavailability can be affected by protein corona formation when biomolecules bind to the surface of NPs during their circulation in blood and CSF, affecting targeting, cellular uptake, and drug release from NPs; in this context, PEGylation can prevent binding between NPs and biomolecules [66]. However, more studies are needed to evaluate the biosafety of NPs in the treatment of CNS pathologies.

Different routes of NP administration, including intravenous, oral, transdermal, and intranasal routes, have been investigated for their ability to reach the brain. Although the intravenous route is invasive, it offers the best onset of activity and bioavailability because it avoids first-pass metabolism. Additionally, intranasal administration provides a less invasive method to overcome the impermeability of the BBB through the entry of NPs into the brain through olfactory neurons. Another method to increase the entry of NPs into the brain is transient opening of the BBB with focused ultrasound. Notably, although NPs have been tested in several preclinical models of neurodegenerative and neurological diseases, they have only been approved for use in multiple sclerosis [67].

### 3.1. Impact of Physicochemical Properties

Achieving optimal performance requires fine-tuning the key physicochemical properties of the NPs. Size ranges generally hover below 200 nm to facilitate endocytic uptake, whereas surface charges can modulate interactions with negatively charged cell membranes. Rigid inorganic NPs may exhibit extended circulation but pose heightened toxicity, whereas soft, polymeric, or liposomal carriers may degrade more readily. Researchers continue to investigate how these parameters affect the ability of NPs to localize within brain tissue without causing undue inflammation or immunological responses [63,64].

The surface charge and shape of NPs influence their ability to penetrate the brain, since positively charged NPs can use adsorptive transcytosis to enter BECs [68], and rod-shaped NPs have demonstrated increased brain uptake [67]. Furthermore, NP size is crucial for their permeability; a smaller size (1–10 nm) facilitates passive paracellular and transcellular transport through BECs, whereas NPs with a size of up to 200 nm are actively transported by carrier proteins, absorptive-mediated transcytosis (AMT), and receptor-mediated transcytosis (RMT) [66]. However, small NPs are the most efficient at penetrating the brain. For example, an evaluation of the ability of gold NPs of different sizes to cross the BBB revealed that only 10 nm-sized NPs accumulate in several rat tissues, including the brain [69]. Similar results were observed using polybutylcyanoacrylate NPs carrying methotrexate, where NPs smaller than 100 nm penetrated the BBB; moreover, in this case, coating the NPs with the surfactant polysorbate 80 improved the brain permeability of the 70 nm NPs [70]. Thus, coating with NPs is a commonly used method to improve their permeability in the brain.

### 3.2. Polymer Nanoparticles

Polymer NPs are formed from either natural or synthetic polymers tailored to specific applications. Common natural materials include proteins and polysaccharides, whereas synthetic options include PLGA, poly(vinyl alcohol) (PVA), and polystyrene (PS). These polymers are typically chosen for their biocompatibility [71], biodegradability, and ease of functionalization [72], enabling them to serve multiple roles, such as stabilizing NP formulations, controlling release kinetics, and facilitating targeted delivery of encapsulated drugs [73].

PLGA, in particular, has garnered significant attention because of its FDA approval and well-documented safety profile [74]. For example, Hu et al. [75] conjugated lactoferrin (Lf) to PEG–PLGA NPs to deliver coumarin-6 in an in vivo model. In a different line of research, Dr. Nitin Joshi and colleagues [76] used PLGA to ferry siRNA across an intact BBB, reducing tau protein levels by 50% in mice, a strategy with potential for AD therapy. Katila et al. [77] reported that Lf–RSV–PLGA–NPs (resveratrol-loaded PLGA NPs functionalized with lactoferrin) markedly improved neuroprotection in a mouse model of MPTP-induced PD.

Although polymeric NPs offer advantages in terms of biocompatibility and versatility, unresolved issues include controlling the particle size during production, ensuring long-term storage stability, and confirming their biosafety at large scales [78]. Addressing these obstacles will be key to advancing polymer NP systems for human application.

### 3.3. Liposome Nanoparticles

Liposomes are spherical structures consisting of one or several phospholipid layers that surround a central aqueous compartment [79]. First conceptualized by Bangham [80], they have been widely applied because of their relatively low toxicity and structural resemblance to biological membranes. The main drawback of simple liposomes is their rapid clearance by the reticuloendothelial system. To mitigate this, researchers modify liposome surfaces with PEG, generating so-called “stealth” liposomes that remain in circulation longer [81]. A notable example is Doxil, a PEGylated liposomal formulation of doxorubicin [82].

In neurological applications, researchers have focused on the potential of liposomes to cross or bypass the BBB and deliver therapeutic payloads directly to diseased tissues. Senapati et al. [83], for example, formulated multifunctional liposomes that target Aβ oligomers and are implicated in AD pathophysiology. Targeting may be achieved passively, through inherent permeability and retention in certain tissues, or actively, by incorporating ligands or antibodies that bind to specific receptors on endothelial or diseased cells [84]. One breakthrough in nucleic acid therapy involves Onpattro (patisiran), a cationic liposomal siRNA formulation that has shown efficacy in tumor models [85].

Further innovations have led to microenvironment-sensitive liposomes that respond to changes in temperature, pH, or oxidative stress. For example, ThermoDox releases doxorubicin upon exposure to hyperthermia [86]. Gong et al. [87] employed pH-sensitive liposomes to treat cerebral hemorrhage, exploiting the acidic extracellular environment near the hematoma for localized drug release. Solid lipid NPs and related nanostructured lipid carriers also hold potential for treating CNS disorders, although improvements in drug loading and stability are needed [88].

### 3.4. Polymer Micelles

Polymer micelles form when amphiphilic polymers, which are molecules that possess both hydrophilic and hydrophobic properties, organize themselves into a core–shell arrangement once they reach a specific concentration known as the critical micelle concentration [89]. The hydrophobic core encloses poorly soluble drugs, whereas the hydrophilic corona stabilizes the micelle in biological fluids [90]. Compared with some NP or liposome production methods, micelle fabrication can be relatively straightforward and amenable to large-scale processes [91].

**Figure 2:**
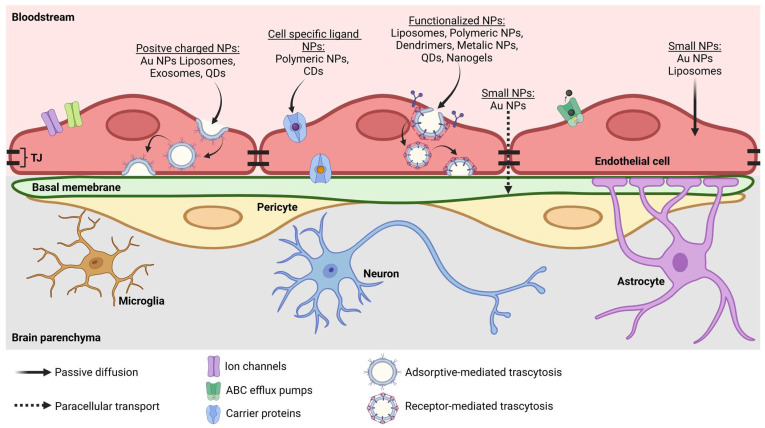
Mechanisms of nanoparticle transport across the blood–brain barrier. NPs can cross or modulate the BBB via different routes: passive diffusion for small NPs with a size of 1–10 nm; transient modulation of TJs to control paracellular permeability; receptor-mediated transcytosis, in which ligands such as transferrin or insulin that bind to receptors on brain endothelial cells are exploited; transport by carriers when functionalized with a specific ligand; and adsorptive-mediated transcytosis, in which positively charged or specialized coatings favor endothelial uptake. Once inside the brain, NPs release therapeutic payloads (e.g., drugs, siRNAs, and growth factors) to target neurons, astrocytes, pericytes, or microglia, potentially restoring BBB function and alleviating neurodegenerative processes. Created in https://BioRender.com (accessed on 12 February 2025).

Drug internalization typically proceeds via endocytosis, after which release occurs through micellar dissociation or diffusion [92,93]. Ongoing challenges include achieving stable micelles in the bloodstream, extending the circulation time, and refining stimuli-responsive systems that release drugs under specific conditions, such as pH changes, redox shifts, or increased temperatures [94]. Innovative designs, such as lactoferrin-conjugated (Lf-PIC@Se) micelles [95] or ROS-activated micelles for ischemic stroke [96], highlight the versatility of this platform in neurological applications, offering neuroprotection and targeted intervention (Figure 2).

### 3.5. Opportunities and Challenges for Nanoparticle Transport Across a Damaged Blood–Brain Barrier

On the other hand, when the BBB is damaged, the entry of some therapeutic molecules into the brain can be facilitated. Traumatic brain injury (TBI) and ischemic stroke are known to induce physical disruption of the BBB and its temporary opening. MRI studies in patients with TBI have shown that BBB permeability spreads to brain areas that are not damaged and can persist during an acute period after TBI or in the long term, from months to years. In the case of ischemic stroke, disruption of the BBB occurs from the beginning of the insult until several weeks later. In addition, biphasic opening of the BBB was observed [18,28]. In this context, opening of the BBB in a model of cerebral artery occlusion in rats was used to increase the brain uptake of PEGylated liposomes loaded with asialoerythropoietin, which accumulate in the ischemic region and decrease neuronal apoptosis [97]. However, it is important to note that BBB opening under pathological conditions may not be generalized throughout the brain. For example, BBB permeability in multiple sclerosis patients was observed to increase only in the white matter. Albumin infiltration during ischemic conditions was more pronounced in the CA1 region than in the CA3 region. In addition, in rats with status epilepticus, BBB leakage increases in specific brain areas, such as the entorhinal cortex, amygdala, and piriform cortex [98].

Experimental methods have been used for the transient opening of the BBB via mechanical, chemical, and thermal stimuli. Focused ultrasound is commonly used to increase BBB permeability and deliver therapeutic agents of different sizes across the BBB. In animal models, 1.5 MHz ultrasound bursts increased BBB leakage to horseradish peroxidase (40,000 Da) and lanthanum chloride (~139 Da), which was associated with the redistribution and loss of the TJ proteins occludin, claudin-5, and ZO-1 [99]. Another study in mice using focused ultrasound in combination with microbubbles revealed that the size of the BBB opening may depend on the intensity of the pulse, since at 0.31 MPa, the size of the BBB opening was less than 3 kDa; at 0.51 MPa, it increased to 70 kDa; and at 0.84 MPa, it reached 2000 kDa [100].

## 4. Using Transcytosis to Selectively Restore and Cross the Blood–Brain Barrier with Nanoparticles

The BBB is a highly specialized portal through which select molecules pass through to reach the brain parenchyma. A key cell type that forms the BBB is BECs, which confer the barrier property of the BBB because their intercellular junctions, namely TJs, seal the free movement of molecules between BECs. Like TJs, BECs are also polarized cells that display different types of membrane proteins in their luminal domain than in their membrane domain, which faces the brain parenchyma [12,101]. Molecules move across BECs through specialized transport proteins such as ion channels or cotransporters [12,102]. In addition to these transport mechanisms, BECs also transport macromolecules via transcytosis, a process through which macromolecules move from the surface domain of one polarized cell to another.

One of the main mechanisms of transcytosis between the milieu and the brain is RMT, which consists in a ligand–receptor interaction that allows the transport of specific macromolecules between two compartments through vesicles covered with clathrins. BECs express various types of receptors that undergo RMT, and some of the most studied receptors for therapeutic purposes are the transferrin receptor (TfR), low-density lipoprotein receptor (LDLR), and insulin receptor (IR). The first two receptors are targeted with antibodies that enhance drug delivery, and one has even reached clinical stages for the treatment of patients with mucopolysaccharidosis type I [103,104]. On the other hand, nonspecific transport of macromolecules usually involves the trafficking of vesicles covered with caveolae [105,106].

Interestingly, the BBB is a dynamic structure that changes as an individual ages, as studies in rodents have demonstrated that its transport properties shift from selective clathrin-mediated transcytosis to nonselective caveolae transport in aged mice [107]. Similarly, transcytosis in AD is widely affected, as genetic variants of genes that participate in the clathrin-mediated transcytosis of Aβ in the BBB, namely *PICALM*, *BIN1*, *CD2AP*, and *RIN3*, are risk factors for this neurodegenerative disease [108,109]. Although alterations in transcytosis represent a challenge and a health issue, they could also represent an opportunity for the efficient design of drug delivery strategies based on NPs not only for crossing the BBB but also for restoring its physiological function in AD.

Lipid NPs are efficient carriers for delivering gene expression vectors that have revolutionized gene delivery in patients who receive vaccines [110]. In this way, NPs could be used to deliver DNA vectors to ectopically express healthy variants of genes related to transcytosis in the BECs of AD patients who carry genetic variants with potential risk for this disease, such as *PICALM*, *BIN1*, *CD2AP*, and *RIN3*. Since lipid NPs have been functionalized with peptides such as transferrin to target the BBB [111], designing lipid NPs carrying DNA plasmids encoding healthy variants of genes related to transcytosis functionalized with peptides to target BECs is a promising therapeutic strategy for treating AD.

Given that one of the main properties of NPs is the protection of peptides from degradation [112], they could be exploited to increase the viability of therapeutic peptides for BECs. Recent evidence indicates that statin treatment is correlated with a decrease in AD risk, thus suggesting that statins are a protective factor against this disease [113]. Since atorvastatin reduces caveolae in endothelial cells, the protective effects of some statins in AD may be at least partially exerted on BECs [114,115]. Therefore, atorvastatin and other statins could be conjugated with NPs functionalized with peptides to target BECs, such as peptides for transferrin or insulin receptors, to test whether this therapeutic strategy could represent a potential treatment for AD. The functionalization of peptides or other types of molecules with NPs is an attractive strategy to enhance their biological effect [112]. Therefore, the biomolecules described above could be protected from degradation with the use of NPs that could be further functionalized with peptides to target receptors that are strongly expressed in BECs, such as insulin or transferrin receptors.

RMT has been employed for drug delivery to the brain by conjugating NPs with ligands or antibodies for receptors expressed on the surface of BECs. For example, the conjugation of albumin NPs with transferrin increased the brain uptake of *3′-azido-3′-deoxythymidine* (AZT) after 4 h of systemic administration in rats [116]. Similar results were observed in mice treated with gold NPs, in which the amount of transferrin bound to the NPs correlated with their ability to bind to their receptor on BECs but also to detach from the receptor and reach the brain side of the BBB [117]. Insulin-conjugated NPs and insulin receptor antibody-conjugated NPs also improved drug delivery to mouse brains after intravenous injection [118].

Peptide conjugation has been used to target receptors in BECs. Compared with nonconjugated NPs, *FITC-dextran-PLGA* NPs conjugated with DAS peptide bind to nicotinic receptors in BECs, increasing NP uptake [119]. In addition, B6, agiopep-2, and G23 peptides that bind transferrin receptors, LRP1, and gangliosides, respectively, have been shown to improve the brain uptake of NPs through transcytosis [68,120,121]. The B6 peptide was used to conjugate NPs loaded with sialic acid, which has been demonstrated to improve memory and learning in AD models [122], and to conjugate NPs carrying epigallocatechin gallate, a compound that prevents α-synuclein aggregation and alleviates PD-like symptoms in mice [123]. Moreover, transferrin receptors are upregulated in BECs under conditions that increase inflammation and ROS levels [124], such as neurodegenerative diseases; in this context, the use of NPs coupled with transferrin can increase their binding to BECs.

Several NP systems focused on AD treatment have been developed to inhibit Aβ production and aggregation. PEGylated NPs loaded with a siRNA for *BACE1*, a key enzyme that produces Aβ, and coupled to the CGN and Tet1 peptides to target the BBB and neurons, respectively, decreased BACE1 levels, Aβ plaques, and phosphorylated tau and improved cognition in *APP/PS1* transgenic mice [125]. In addition, since glutathione is transported through the BBB, PEGylated liposomes conjugated with glutathione were tested in *APP/PS1dE9* transgenic mice to introduce antibody fragments to bind Aβ in the brain [126]. Drug combinations have also been employed to treat AD. Albumin NPs modified with transcriptional activator protein (TAT) and *monosialotetrahexosylganglioside* (GM1, a lipid that enhances brain access), coloquinol (a metal-ion chelator), and donepezil (an acetylcholinesterase inhibitor) restored the balance between Aβ aggregates and acetylcholine and improved spatial learning and memory after 30 days of intranasal administration [127].

On the other hand, glial-derived neurotrophic factor (GDNF) was found to have a beneficial effect on PD. Lactoferrin-coupled NPs were used for the delivery of GDNF to the brain of a 6-OHDA PD model in rats, and five systemic doses of lactoferrin-NPs produced a potent neuroprotective effect in the animals [128].

## 5. Nanoparticles to Target the Neurovascular Unit

**Figure 3:**
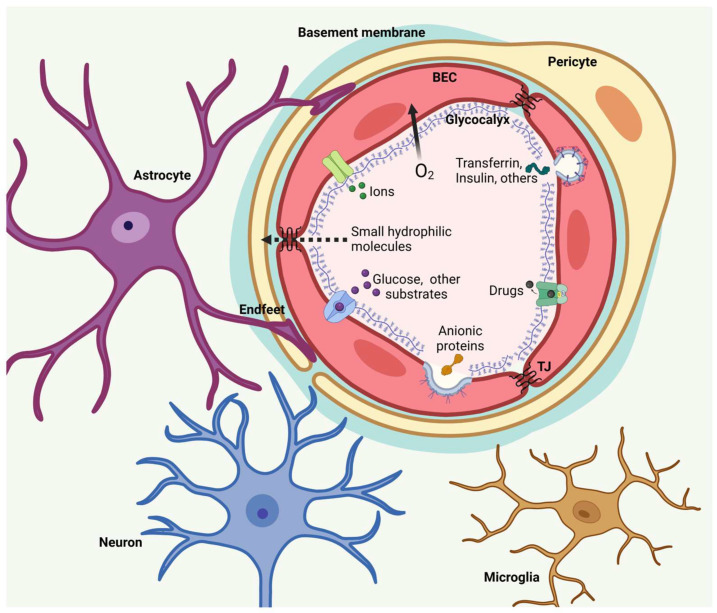
Composition of the neurovascular unit. Brain endothelial cells (BECs) form a barrier that separates the bloodstream from the brain parenchyma. In brain capillaries, BECs are closely connected through tight junction (TJ) proteins that restrict paracellular transport. BECs are surrounded by pericytes and make contact with astrocytic endfeet that maintain the integrity and function of the BBB. In addition, a basement membrane secreted by NVU cells covers brain capillaries that are lined by the glycocalyx on the luminal surface. Solute transport through BECs can occur via passive diffusion (arrow), paracellular transport (dashed arrow), ion channels, carriers, ABC transporters, and absorptive-mediated transcytosis. Created in https://BioRender.com (accessed on 12 February 2025).

The basic component of the BBB is the neurovascular unit (NVU), which is a complex structure composed of BECs, glia, and neurons associated with a basement membrane secreted by NVU cells and an enriched matrix of proteoglycans, glycoproteins, and glycosaminoglycans (glycocalyxes) that covers the luminal surface of BECs [12]. BECs in the NVU strictly control the passage of substances between the brain and the blood, thus protecting the CNS from neurotoxic substances while also ensuring metabolic needs (Figure 3).

The maintenance of the NVU and BBB integrity and function is complex and requires multiple signaling pathways. Wnt signaling is a key element in NVU integrity and BBB function since astrocyte-derived Wnt proteins maintain the morphology of astrocytic endfeet, low BBB permeability and caveolae vesicular transport, and correct expression of cadherin 5 and the TJ accessory protein ZO-1 in BECs. Interestingly, this morphogen does not contribute to the integrity of TJs or high transendothelial electrical resistance (TEER) [129]. On the other hand, Shh maintains BBB impermeability in a complementary way to Wnt, as it increases the TEER, which is correlated with an increase in TJ proteins such as claudin-5 and occludin in BECs [130]. Furthermore, Tie-2 signaling also modulates NVU and BBB properties through angiopoietin 1 (Ang 1) and 2 (Ang 2), which promote and inhibit this signaling pathway, respectively [131]. Moreover, Ang 1 and Tie-2 are present in healthy BECs, and their expression is downregulated after cerebral vessel injury; in contrast, Ang 2 expression increases after cerebral vessel damage [132]. In line with this evidence, the ectopic activity of Ang 2 increases BBB leakage and caveolae transport, impairs pericyte and astrocytic endfeet integrity, and decreases TEER. Notably, pharmacological activation of Tie-2 signaling rescues TEER impairment in vitro and prevents BBB leakage in vivo. Together, this evidence indicates that Ang 1 and 2 decrease and increase BBB permeability, respectively.

Mitochondrial activity plays a key role in the maintenance of the NVU, and many mitochondria are located in astrocytic endfeet to buffer cytosolic calcium and produce the ATP necessary for neurovascular coupling. Mitochondrial calcium homeostasis is modulated by the transporters MCU_CX_ and NCLX, which mediate its uptake and efflux, respectively. The inhibition of MCU_CX_ and activation of NCLX promote NVU repair in ischemic stroke via the use of NPs for the delivery of retinoid X receptor and thyroid hormone receptor agonists, which, through transcriptional mechanisms, induce mitochondrial biogenesis, leading to a reduction in inflammation and promoting the proliferation of BECs and glial cells [124]. Therefore, MCU_CX_ and NCLX could be potential therapeutic targets for nanomedicine in neurodegenerative diseases. In addition, the high density of mitochondria in astrocytes and BECs makes them prone to oxidative damage, and biodegradable NPs functionalized with triphenylphosphonium cations have been used to target mitochondria in the brain and deliver antioxidant agents that mitigate oxidative damage in astrocytes and promote neuronal protection [133].

The conjugation of NPs with different molecules is widely used to increase their permeability and tissue selectivity. The presence of glucose transporter 1 (GLUT1) in the BEC membrane has been used to develop glucose-conjugated NPs; for example, 4 nm glucose-coated gold NPs can selectively cross the human brain endothelium and accumulate in astrocytes in an in vitro three-dimensional model of the BBB [134]. However, the use of glucose-conjugated NPs is feasible when GLUT1 is overexpressed, such as in brain tumors [135,136].

Many neurodegenerative diseases are characterized by central hypometabolism, which has been attributed in part to the reduced expression of GLUT1 and GLUT3 in BECs and neurons, respectively [137]. In AD, reduced levels of both transporters have been widely described in the hippocampus and cerebral cortex of patients and rodent models [138]. This was also observed in the caudate nucleus of postmortem tissue from patients with Grade 3 Huntington’s disease [139]. In PD, GLUT1 expression is controversial since some authors have reported decreased GLUT1 levels in the striatum of animal models, whereas others have reported no changes [137]. In AD, reduced expression of GLUT1 and GLUT3 is related to downregulation of hypoxia-inducible factor 1 (*HIF-1*). Conversely, liraglutide was shown to be effective in restoring glucose uptake in the BBB in AD patients [37]. These findings suggest that NPs can be used for the delivery of *HIF-1* mRNA or liraglutide to BECs and improve glucose metabolism in the brain.

Annexin A1 in BECs prevents leukocyte migration, possibly through the regulation of tight and adherens junctions, since alterations in occludin and VE-cadherin were observed after the deletion of annexin A1 in mice [140]. Agrin is a proteoglycan present in the basal membrane of the NVU whose expression is abundant in brain areas with AB plaques and in the NFTs of AD patients, suggesting a role for this protein in the aggregation of Aβ and tau [141,142]. These findings suggest the use of annexin A1 and agrin as targets for NP systems in BECs and the basal membrane. In addition, it is important to consider that the basement membrane itself can represent a filter for the retention of NPs since the anionic sites present in the basement membrane and on the surface of BECs can influence the interaction of negatively charged NPs with basement membrane proteins [26].

Recently, heterocellular spheroids that resemble the NVU were developed via the self-assembly of human BECs, brain vascular pericytes, and astrocytes combined with primary cultures of neurons and microglia from neonatal rats. These spheroids expressed the markers associated with each cellular component and presented a solid structure surrounded by endothelial cells expressing adherens junctions. Furthermore, this model was developed to study the interaction between NPs and the NVU and revealed that chitosan polymeric NPs (92–463 nm) accumulate mainly on the surface of the spheroid, whereas gold NPs (10 nm) cross the surface of the spheroid (BECs) and are taken up by microglia. Interestingly, graphene NPs (5 and 10 nm) significantly damaged the spheroid structure [143]. These results highlight the importance of developing NVU models not only to test whether NPs can cross the BBB but also to restore it as a fundamental strategy for the treatment of neurodegeneration (Table 1).

NP-based therapies offer an innovative strategy to treat neurodegenerative diseases by enhancing drug delivery, bypassing the BBB, and reducing inflammation. Nevertheless, a comprehensive understanding of their long-term efficacy and potential adverse effects is crucial for assessing their feasibility for clinical use.

Although many NPs show promise in providing sustained drug release and neuroprotective effects, the durability of these outcomes remains uncertain. For example, nanogels and liposomes have demonstrated the ability to stabilize disease progression through controlled drug delivery and immune regulation in preclinical models. Similarly, dendrimers prolong the inhibition of neurotoxic protein aggregation in AD and PD models [144]. However, translating these findings to clinical practice will require further validation to confirm their long-term effectiveness. Achieving consistent therapeutic benefits depends largely on refining drug release mechanisms, NP degradation rates, and targeting accuracy.

The safety profile of NPs is closely related to their characteristics, including size, composition, and surface properties. Prolonged exposure to certain NPs can activate the immune system within the CNS, leading to neuro-inflammation [145,146]. This may exacerbate disease symptoms, as activated microglia release inflammatory cytokines such as TNF-α and IL-1β [147]. Furthermore, metallic NPs, such as those containing iron oxide or gold, can accumulate in brain tissue, increasing the risk of long-term toxicity by disrupting mitochondrial function and triggering oxidative stress in neurons [148].

Several strategies have been employed to reduce these risks. Coating NPs with biocompatible materials, such as PEG, has been shown to minimize immune activation and prolong circulation [149]. NPs less than 100 nm in size are more easily cleared from the body, lowering the risk of bioaccumulation [150]. Additionally, functionalizing NPs with brain-targeting molecules enhances their ability to cross the BBB and limits systemic side effects [151]. Despite these advances, further long-term clinical studies are necessary to evaluate the risks of chronic inflammation, oxidative damage, and NP retention.

The regulatory and ethical considerations for the clinical translation of NP-based therapies are complex and multifaceted. Regulatory agencies require comprehensive data on safety, toxicity, and pharmacokinetics to approve such therapies for human use. Ethical concerns include informed consent, particularly regarding long-term unknown risks, and ensuring equitable access to advanced nanomedicines. Transparency in clinical trial design and data reporting is essential to maintain public trust and regulatory compliance. Additionally, the lack of harmonized international standards on nanotechnology regulation presents challenges for the global adoption of these therapies.

NP-based therapies also hold significant potential for personalized medicine approaches. By tailoring NPs to individual patient profiles, such as genetic markers, disease subtypes, or drug metabolism characteristics, treatments can become more precise and effective. Advances in nanodiagnostics and theranostics enable real-time monitoring of disease progression and treatment response, allowing dynamic adjustments to therapeutic strategies [148]. Personalized NP platforms may also reduce adverse reactions by ensuring more targeted delivery of drugs, ultimately improving treatment outcomes and patient safety [152].

**Table 1:** Types of nanoparticles designed to target the neurovascular unit. Liposomes and polymeric NPs are particularly versatile for brain targeting because of their ease of surface modification and relatively favorable safety profiles. PLGA, poly(lactic-co-glycolic acid); PEG, polyethylene glycol; SLNs, solid lipid nanoparticles.

Nanoparticle Type	Composition (Examples)	Advantages	Challenges	Relevant Applications	References
Polymeric NPs	PLGA, chitosan, PEG-PLGA	Biodegradable, tunable drug release, various functionalization possibilities	Batch-to-batch size control, potential aggregation	siRNA delivery for Alzheimer’s (BACE1 inhibition), PD neuroprotectants	[125,153,154,155]
Lipid-Based (Liposomes, SLNs)	Phospholipids, solid lipids (SLNs)	Biocompatibility, flexible payload loading, stealth via PEGylation	Possible rapid clearance, stability issues	Aβ-targeted liposomes, curcumin-loaded SLNs for neuro-inflammation	[83,156,157,158]
Micelles	Amphiphilic block copolymers (PEG-PLA)	Simple to formulate, enhanced solubility of hydrophobic drugs	Potential micelle dissociation in circulation	Lactoferrin-conjugated micelles for targeted PD therapy	[75,77,95,128]
Metallic NPs	Gold (Au), silver (Ag), iron oxide	Easy to image (MRI, CT), magnetic targeting (Fe_3_O_4_), large surface area	Risk of toxicity, accumulation long term	AuNPs inhibiting Aβ aggregation, magnetic NPs guiding stem cells	[159,160,161,162,163]
Carbon-Based	Graphene, carbon nanotubes (CNTs)	High surface area, mechanical strength, suitable for loading multiple agents	Potential toxicity, complex functionalization steps	CNTs as scaffolds for neuronal repair, graphene for reducing oxidative stress	[164,165,166,167]

## 6. Nanoparticle Targeting Tight and Adherens Junctions

**Figure 4:**
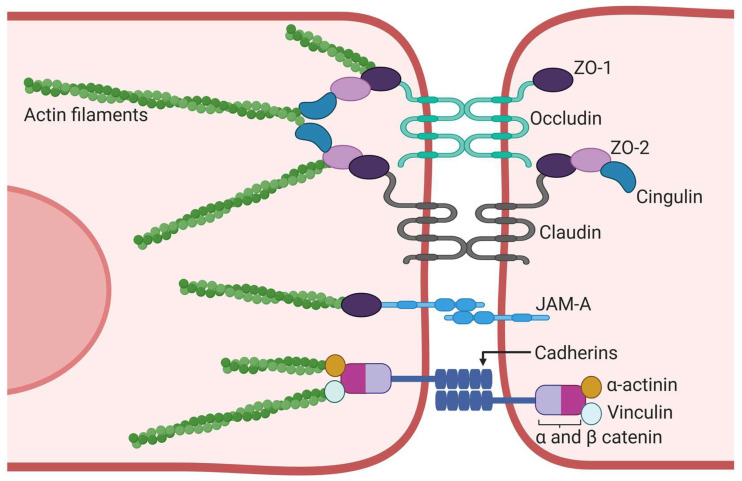
Tight and adherens junctions in brain endothelial cells. Tight junctions are formed by occludin and claudins, which are transmembrane proteins that bind to the scaffolding protein zonula occludens (ZO-1/2), providing a link to Cingulin and the actin cytoskeleton. Junctional adhesion molecule A (JAM-A) also associates with ZO proteins. Adherens is formed by cadherins that form complexes with α-catenin, β-catenin, vinculin, and α-actinin to bind actin filaments. Created in https://BioRender.com (accessed on 12 February 2025).

The main structural components of BECs responsible for their barrier properties are the TJs formed by the integral transmembrane proteins occludin and claudin, and the scaffolding proteins zonula occludens (ZO), cingulin, and AF6. Additionally, adherens junctions (AJs) contribute to barrier integrity and are composed of cadherins, catenins, vinculin, and actin (Figure 4) [13,168,169,170].

TJs are located at the most apical region of cell–cell contacts and play a key role in building the endothelial barrier and maintaining the endothelial polarity of the BBB. TJs regulate the paracellular diffusion of solutes by forming a continuous and impermeable barrier, which allows the supply of water, oxygen, and solutes such as electrolytes but limits the permeability of other molecules such as drugs and xenobiotics through the intercellular space [13,169,171,172,173]. Therefore, the integrity of TJs determines the paracellular permeability of water-soluble molecules across the BBB. In addition, TJs play important roles in cell signaling under physiological and pathological conditions by modulating their expression, subcellular redistribution, and post-translational modifications, which in turn affect protein–protein interactions [13,168,172].

AJs are specialized cell–cell interactions formed primarily by cadherins and catenins that are directly linked to actin filaments. Cadherins regulate endothelial function by activating phosphatidylinositol 3-kinase signaling, which organizes the cytoskeleton and allows the formation of complexes with the VEGF receptor. This cadherin-mediated signaling is essential for the integrity of the endothelial layer and the spatial organization of new blood microvessels [169,174]. In the absence of AJs, endothelial cells cannot form a structured cell layer, and TJs cannot be formed since AJs induce the expression of TJ proteins [171,173,174].

Since the integrity of TJs in the BBB is compromised in neurodegenerative diseases such as AD, PD, multiple sclerosis, and other neurodegenerative diseases [24,175,176], TJs represent potential therapeutic targets that could be addressed by the use of multiple molecules capable of restoring the physiological condition of TJs. For example, activation of Wnt signaling restores the expression of claudin-5 and the adaptor protein ZO-1, which decreases the leakage induced by Aβ [24], suggesting that the Wnt peptide could be a promising protein to overcome BBB impairment. In line with this evidence, caffeine restores impermeability and the expression of ZO-1 in the BBB in an AD mouse model [177].

Claudin-5 is crucial for maintaining BBB integrity since claudin-5 knockout mice die one day after birth [171]. Furthermore, the FD7 peptide, which binds claudins 1 and 5, enhances ionic permeability in a BBB model [178]. Additionally, VEGF secreted by glioblastoma cells plays an important role in increasing BBB permeability by significantly decreasing the expression of claudin-5 in a dose-dependent manner. Conversely, blocking the VEGF/VEGFR pathway with axitinib reduced BBB permeability and improved barrier integrity [175]. These results suggest that the modulation of claudin expression in BECs can be used in nanomedicine for both sealing and permeation of the BBB.

The phosphorylation state of claudins has been implicated in the regulation of paracellular permeability and depends on the type of stimulus (e.g., inflammatory cytokines, oxidative stress, or growth factors). Claudin-5 can be phosphorylated at threonine residues, whereas occludin is phosphorylated at serine and threonine residues, which controls the intracellular distribution of these TJ proteins and the subsequent properties of the barrier [13]. The phosphorylation of occludin and ZO-1 may regulate the assembly or disassembly of TJs [172]. In addition, PP1 and PP2A Ser/Thr phosphatases directly dephosphorylate TJ proteins, leading to increased permeability [168,172]. The kinase PKCζ has a protective effect on TJ integrity since the treatment of endothelial cells with gold NPs leads to increased paracellular permeability by impairing the interaction between occludin and ZO-1 via a mechanism dependent on PKCζ phosphorylation, whereas treatment with arachidonic acid, an activator of PKCζ, partially restores TJ integrity in endothelial cells. Conversely, the phosphorylation of claudin-5 induced by TGF-β1 and RhoK increases endothelial permeability in the brain [171]. Occludins are also phosphorylated by RhoK in brain tissue, which affects TJ function in the BBB. In addition, recent research has shown that gold, silver, and silica NPs can interact with key kinases, affecting signaling pathways and potentially increasing BBB permeability by disrupting TJs [171]. Therefore, changing the phosphorylation state of TJs with NPs may be a feasible way to modulate BBB permeability.

Drug absorption through the paracellular route is generally hindered by drug size and charge. Materials that can stimulate the reorganization of TJs have been identified. To improve paracellular drug transport, TJ modulators have been studied [170,171]. The biopolymer chitosan is a nonspecific permeation enhancer of TJs, whose proposed mechanisms to increase permeability include alterations in cell membrane lipids and interactions with the components of TJs [171]. NPs made of chitosan or its derivatives enhance paracellular permeability and the pharmacokinetics of encapsulated molecules [168]. For example, chitosan NPs optimize the delivery of rivastigmine, a reversible acetylcholinesterase inhibitor used to treat AD [173].

Recent research has focused on the development of materials that temporarily affect the organization of TJ proteins. NPs modified with the BV11 antibody, which targets junctional adhesion molecule A (JAM-A) proteins in TJs, need to be activated by laser stimulation to increase the permeability of the BBB by generating mechanical waves that alter TJs, thus facilitating the delivery of antibodies for treatment [168]. Magnetic iron oxide NPs and titanium oxide nanomaterials affect the function of TJs through a mechanism dependent on VE-cadherin. In addition, silver NPs, gold NPs, and nanodiamond structures can cause the downregulation and dysfunction of TJ proteins, increasing BBB permeability and improving drug delivery. Piezoelectric nanomaterials, which are activated by physical stimuli such as ultrasound, are being studied for their ability to treat CNS disorders and facilitate drug delivery to the brain. However, more research is needed on the effects of ultrasound and the elimination of NPs to avoid long-term damage [168].

## 7. Nanoparticles to Reduce Neuro-Inflammation

Inflammation is one of the mechanisms involved in the body’s defense, which protects organisms from injury, infection, or toxins [179]. It is aimed at removing noxious stimuli and initiating the healing process. The persistence of the inflammatory response leads to altered cell function.

In the CNS, microglia are the primary sensors of noxious stimuli [180]. These cells actively surveil and can sense the state of the brain parenchyma, ensuring that it is maintained within the homeostatic conditions needed to guarantee the proper function of the neuronal circuits [181]. The microglial response to injury implies a change in their function from a surveillance state to a reactive state in which they can migrate, proliferate, and recruit and modulate the activity of other cells, including blood-borne immune cells, neurons, and nonneuronal cells, by releasing cytokines and chemokines in a process known as neuro-inflammation.

Neuro-inflammation leads to the release of the pro-inflammatory cytokines TNF-α and IL-1β from microglia, which increases BBB permeability [182]. Interestingly, microglial activation in the brains of AD-transgenic mice was attenuated by the use of zwitterionic poly(carboxybetaine) (PCB)-based NPs, which were able to decrease the secretion of pro-inflammatory mediators and brain-derived neurotrophic factor (BDNF) and increase Aβ phagocytosis by microglia, reducing Aβ levels, neuro-inflammation, neuronal damage, and memory deficits [183].

During inflammatory conditions such as neurodegenerative disease, activated BECs switch to a pro-inflammatory phenotype that produces thrombin, IL-1β, IL-6, TNF-α, and ROS [12] and overexpresses adhesion molecules involved in leukocyte diapedesis that perpetuate neuro-inflammation. The ability of the overexpression of vascular cell adhesion molecule-1 (VCAM-1) in inflammatory BECs to generate VCAM-1-coupled lipid NPs loaded with thrombomodulin mRNA to eliminate brain edema in a TNF-α-induced acute brain inflammation model in mice was studied [184]. E-selectin is an adhesion molecule that binds neutrophils and is overexpressed after an inflammatory stimulus in human brain microvascular endothelial cells [185]. In line with this evidence, activated endothelial cells overexpressing TNF-α were targeted via anti-E-selectin immunoliposomes loaded with rapamycin to inhibit the proliferation and migration of endothelial cells [186]. Interestingly, the serum protein clusterin ameliorates the pro-inflammatory phenotype of BECs in a rodent model of AD, which suggests that this molecule could be useful in AD treatment by targeting the BBB [187]. This scenario is suitable for testing NPs that can attenuate BBB damage caused by activated BECs.

Astrocytes are also known to respond to noxious stimuli. These cells support and regulate tissue-resident immune cells [188]. During inflammation due to pathological states or injury, astrocytes become reactive, which involves morphological, molecular, and functional changes to recover brain homeostasis. Astrocytes establish a bidirectional interaction with microglia [189]. Under physiological conditions, astrocytes secrete interleukin-33 (IL-33), which promotes microglial synaptic protein phagocytosis [190]. However, after an inflammatory insult, astrocyte–microglia interactions are necessary to trigger the immune response in astrocytes. For example, microglia recognize the bacterial cell wall endotoxin lipopolysaccharide (LPS) via toll-like receptor 4 (TLR4), a receptor that is absent in astrocytes. After activation of the TLR4 downstream signaling pathway, microglia secrete IL-1α, TNF-α, and C1q, which bind with their receptors in astrocyte cell membranes to promote the synthesis of soluble molecules that can drive apoptosis in mature neurons and oligodendrocytes, recruit other immune cells, and even change BBB permeability [191].

Neurodegeneration and neuropsychiatric disorders are the most common consequences of neuro-inflammation [192,193,194]. AD is characterized by the accumulation of Aβ plaques and NFTs formed by hyperphosphorylated tau [18]; however, neuro-inflammation triggered by these pathological proteins is an important component that ultimately leads to neuronal dysfunction and death [195,196]. Consequently, some therapeutic strategies are aimed at reducing central inflammation and therefore promoting neuroprotection. RMT-based strategies permit a uniform distribution of drugs within the CNS owing to the generalized expression of targeted receptors in the BBB [197]. In AD, increased expression of RAGE in the endothelial cells of the NVU was observed near lesion sites [198,199,200,201,202], which also presented increased levels of ROS [203]. The RAP peptide, a ligand of RAGE, has been employed to specifically target NPs at AD lesion sites. Treatment with NPs loaded with ibuprofen and FK506 (Ibu&FK@RNPs), which specifically target RAGE and ROS, improved cognition and reduced Aβ plaques, neurotoxicity, and neuro-inflammation in an AD mouse model.

The use of liposomes is one of the strategies aimed at transporting medications across the BBB [204]. This is achieved by integrating surface-active ligands that promote transcytosis and cationic liposome absorption into the BBB. Liposomes can be coated with glucose, mannose, and cell-penetrating peptides that can carry compounds with antioxidant, anti-inflammatory, and anticancer properties [205]. Curcumin-loaded liposomes have been employed since their lipid composition is similar to that of the outer membrane of exosomes and oligodendroglial precursor cells [206]. Preincubation of SH-SY5Y and BV2 cultures with curcumin-loaded exo-liposomes improved cell viability when they were exposed to the oxidative stress inducer tert-butyl hydroperoxide (t-BHP) [207]. Curcumin has also been loaded into gelatin NPs (Cur@GAR NPs) modified with rabies virus glycoprotein to target neurons to treat ischemic stroke, resulting in a reduction in nerve damage and improved cognitive performance [208].

Nanomedicines have also sought to target specific cell populations in the CNS. This technology relies on the ligand–receptor interactions of each cell type during neuro-inflammation [66]. Targeting endothelial cells to reduce immune cell trafficking has been achieved by preventing endothelial overexpression of adhesion molecules [209]. Magnetic resonance imaging using antibody-coupled microparticles has been used to monitor leukocyte recruitment to the brain in multiple sclerosis and to prevent leukocyte infiltration. This process requires the expression of adhesion molecules in the BEC membrane, such as P-selectin, ICAM-1, and VCAM-1 [210,211]. This strategy restricts leukocyte infiltration with the antibody natalizumab, which prevents lymphocyte diapedesis mediated by VCAM-1. Moreover, elevated VCAM-1 levels were also observed in the brain endothelium of AD mice [211], suggesting that neuro-inflammation mediated by leukocyte infiltration could be attenuated via the use of antibody-conjugated NPs that block VCAM-1, P-selectin, and ICAM-1 in the brain vasculature.

Neurons are important therapeutic targets since their function becomes impaired during disease. Therefore, maintaining their homeostatic function and promoting survival has been a priority in the search for therapeutic strategies for neuro-inflammation. For example, a novel drug delivery system to reduce edema in stroke was developed by using glyburide-loaded NPs coated with the membrane of neural stem cells (NSCs) overexpressing the chemokine receptor CXCR4 to bind SDF-1, which is enriched in the ischemic brain. This approach improves pharmacological treatment, resulting in increased mouse survival and a reduction in infarct volume [212].

**Figure 5:**
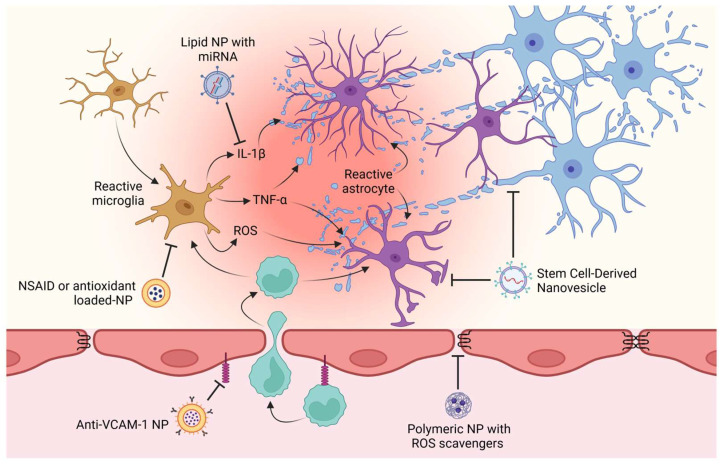
Nanoparticle-based strategies to counteract neuro-inflammation. During neuro-inflammation, microglia switch to an active phenotype in which they secrete pro-inflammatory cytokines such as IL-1β, TNF-α, and reactive oxygen species (ROS), leading to astrocyte activation. Moreover, inflamed brain endothelial cells express cell adhesion molecules that facilitate the infiltration of peripheral immune cells, thereby exacerbating inflammation that damages the BBB and induces neuronal death. Therefore, some proposed NP-based strategies to reduce neuro-inflammation include the use of NPs functionalized with neutralizing antibodies against cell adhesion molecules such as VCAM-1; the use of NPs for the delivery of anti-inflammatory compounds (e.g., nonsteroidal anti-inflammatory drugs (NSAIDs)) or antioxidants to prevent microglial activation; the use of NPs loaded with miRNAs to inhibit the expression of pro-inflammatory cytokines; the use of NPs loaded with ROS scavengers for the restoration of tight junctions in the BBB; and the use of stem cell-derived nanovesicles to attenuate gliosis and improve neuronal regeneration. Arrows indicate release or activation, while blunt arrows represent inhibition. Created in https://BioRender.com (accessed on 17 February 2025).

NP characteristics can improve drug pharmacokinetics, enabling them to overcome the main problems associated with neuro-inflammatory treatments. The efficacy of using NPs to treat neuro-inflammation in animal models has proven to be an effective strategy that continues to be explored and perfected in different models, intending to eventually be implemented in clinical protocols (Table 2 and Figure 5).

## 8. Nanotechnology, Stem Cells, and the Blood–Brain Barrier in Neurodegenerative Diseases

### 8.1. Stem Cell Delivery

Overcoming the challenges posed by the BBB is crucial for advancing therapies for neurodegenerative diseases. Addressing this requires an understanding of key aspects, such as stem cell delivery mechanisms, homing strategies, and the role of NPs in enhancing therapeutic outcomes. To delve into these topics, it is essential to provide insights into innovative approaches for CNS repair. Taken together, these findings offer a comprehensive framework for developing effective strategies to address BBB permeability and damage in neurodegenerative conditions.

Stem cells offer a unique regenerative approach to treating neurological disorders, defined by their capacity for self-renewal and the potential to differentiate into various specialized cell types [218]. In particular, induced pluripotent stem cells (iPSCs) can be guided to become neurons that are pertinent to PD or other cell types necessary for CNS repair [219]. Additionally, mesenchymal stem cells (MSCs) may be genetically engineered to produce or carry therapeutic molecules, delivering them to sites of damage in the brain or spinal cord [220,221].

### 8.2. Mechanisms of Stem Cell Homing

MSCs exhibit an innate “homing” mechanism guided by inflammation-related signals, allowing them to traverse vascular endothelial barriers under certain conditions [222,223]. They secrete cytokines, growth factors, and miRNAs that stimulate tissue repair and modulate immune responses [216,217]. Delivery methods vary, including direct intracerebral transplantation, intravenous injection, and intranasal routes, each with distinct advantages and limitations regarding targeting precision and invasiveness [224,225]. Early clinical trials suggest encouraging safety profiles, with minimal adverse events [226].

### 8.3. Nanoparticle-Based Enhancements

Nanotechnology adds another dimension to stem cell therapies by enhancing targeting, tracking, and overall efficacy [227]. Magnetic NPs, for example, facilitate the sorting and labeling of stem cells, whereas quantum dots enable real-time imaging of the cell distribution. Exosomes, nanovesicles secreted by cells, have the ability to cross the BBB and target pathological brain regions. Using gold NP labeling and X-ray computed tomography, intranasally administered exosomes derived from bone marrow MSCs (MSC-exos) were tracked in murine models of stroke, autism, PD, and AD. MSC-exos selectively accumulated in inflamed regions of pathological brains up to 96 h post-administration, which correlated with neuro-inflammatory signals. Notably, neuronal cells in these areas preferentially take up MSC-exos, whereas healthy controls exhibit diffuse migration and clearance by 24 h [161]. These findings highlight the therapeutic potential of MSC-exos for targeted drug delivery in neurodegenerative and neuro-inflammatory conditions. NPs can also promote cellular functions such as proliferation and differentiation through controlled interactions at the cell membrane or intracellularly. Gold-based nanoformulations targeting NSCs in neurogenic niches show promise for brain repair. Gold nanorods (Au NRs) conjugated with medium-density transferrin peptides cross the BBB effectively and, when activated with near-infrared light, accumulate in NSC niches [163]. These results demonstrate the potential of tailoring NP properties for targeted neuroregeneration. In tissue engineering, decellularized scaffolds may be repopulated with iPSCs to form functional organoids, potentially addressing organ failure and offering new platforms for studying brain disorders [228]. In addition, nanotoxicology research is pivotal to confirm that these materials are safe for clinical applications [227].

### 8.4. Nanoparticles in Stem Cell Regeneration

NPs have emerged as promising adjuncts in stem cell therapy for AD, as they use carriers such as polymers, metals, lipids, carbon nanotubes, and solid lipid biomaterials [165]. One noteworthy example involves superparamagnetic iron oxide NPs (SPIOs), which enable targeted delivery through magnetic guidance [229]. When conjugated with retinoic acid or combined with dextran sulfate and polyethyleneimine, these particles can increase the proliferation of human embryonic progenitor cells (hEPCs) by up to 83-fold relative to that of retinoic acid alone [230]. In MSCs, SPIO-ferucarbotran has been shown to increase cyclin D1 and CDK-4 levels while reducing the level of intracellular H2O2 [231], thus stimulating healthy cell growth.

Biodegradable poly(β-amino ester) NPs similarly aid in the gene transfection of embryonic stem cells (ESCs), safeguarding their viability and pluripotency [232]. ZAAM is a novel NP system that combines antisense oligonucleotide (ASO) therapy with NSC membrane (NSCM) coatings and an aptamer (Apt 19S) for targeted PD treatment. ZAAM enables systemic delivery of ASOs, overcoming the invasiveness of intrathecal administration, whereas NSCM facilitates BBB penetration and NCS recruitment to regenerate dopaminergic neurons. Behavioral tests confirmed enhanced ASO efficacy and targeted therapeutic outcomes [233]. This innovative approach highlights biomimetic, BBB-penetrable drug carriers as promising tools for the precise diagnosis and treatment of neurodegenerative diseases such as PD. Curcumin-loaded PLGA NPs additionally drive Wnt signaling in NSCs, enhancing neuronal proliferation, differentiation, and memory-related functions in AD models [215]. Notably, NP sizes in the 20–70 nm range often prove optimal for encouraging stem cell differentiation [234], and materials such as graphene or graphene oxide can efficiently direct iPSC differentiation [235]. Moreover, gold NPs help reduce oxidative stress in AD by modulating the NF-κβ and mi-R-21-5p pathways [162]. Taken together, these findings illustrate the growing synergy between nanotechnology and stem cell platforms for CNS repair.

### 8.5. Mechanisms Behind Stem Cell Blood–Brain Barrier Transmigration and Homing

Different stem cell populations (mesenchymal, neural, embryonic, and iPSC) have been proposed for treating neurodegeneration [236,237,238]. MSCs stand out for their capacity to secrete trophic and immunomodulatory factors that shield injured tissues and encourage their differentiation [239,240]. Traumatic events such as stroke or TBI often transiently compromise the BBB, paving the way for MSC migration through paracellular pathways [241,242], a process further supported by MSC-driven loosening of TJs [243,244].

Once in the circulation, MSCs follow a series of steps (tethering, rolling, adhesion, transmigration, and migration) that are regulated by chemokines and adhesion molecules (e.g., integrins such as VLA-4 bind to endothelial VCAM-1) [213,214]. The secretion of matrix metalloproteinases (MMPs) then degrades basement membranes, facilitating deeper penetration into the CNS [245]. Although local injection (e.g., intracerebral) ensures increased targeting efficiency, systemic administration (e.g., intravenous) may be more practical, albeit less direct, as cells can become trapped in filtering organs such as the lungs [246,247,248,249]. The intranasal and intramuscular routes show potential in specific scenarios, but consensus on the optimal method for delivering stem cells to the CNS remains elusive [250,251,252].

### 8.6. Stem Cell Therapy in Neurodegenerative Diseases: Mechanisms, Benefits, and Challenges

The pathological role of the BBB in conditions such as AD, PD, and ALS underscores the difficulties of delivering effective treatments [253]. Stem cell therapy has steadily gained traction as a potential avenue for CNS repair [254], with MSCs and NSCs often investigated for their ability to slow or even halt neurodegeneration [255,256]. Through paracrine signaling, MSCs release growth factors, cytokines, and neurotrophic molecules that activate local repair pathways [257]. Although complete neuronal replacement by transplanted cells appears limited, partial functional recovery has been reported [258,259]. In rodent models of TBI, when MSCs are administered intravenously, they can differentiate into neuron- or astrocyte-like cells, promoting enhanced motor and sensory function [242,260]. Notably, clinical investigations of pediatric TBI suggest improvements in Glasgow Coma Scale (GCS) scores, albeit with variability [261]. Likewise, in PD, MSCs may increase dopaminergic neuron viability, and clinical trials have reported satisfactory safety profiles over extended periods [262,263]. The control of MSC-driven inflammation may also help restore BBB function [264,265]. In addition, in AD, bone marrow-derived MSCs in mouse models can decrease amyloid-β levels by promoting microglial activation, autophagy, and clearance mechanisms [266,267]. Phase I clinical data for umbilical cord MSCs indicate good tolerability, although larger trials are needed to evaluate their efficacy [268]. Finally, in the context of ALS, engineered MSCs that secrete neurotrophic factors have slowed motor neuron loss and partially improved motor performance in preclinical models, with some positive signals in early-phase clinical studies [269,270].

Despite optimism, multiple hurdles remain, including the route of administration, long-term safety, and elucidation of how MSCs or iPSCs exert neuroprotective effects [271,272]. Standardized methods and rigorous clinical designs are crucial to clarify these issues.

### 8.7. Stem Cell Models for the Blood–Brain Barrier: Development and Maintenance

Emerging evidence shows that during embryogenesis, neural progenitor cells (NPCs) can induce BBB phenotypes in brain microvascular endothelial cells (BMECs) before astrocytes are fully established. For example, coculturing rat cortical NPCs (from embryonic day 14) with adult rat BMECs increased transendothelial electrical resistance (TEER) and increased the expression of TJ proteins (claudin-5, occludin, and ZO-1) [273,274]. Wnt signaling seems critical in this early developmental phase [275,276].

To investigate human BBB development in vitro, researchers have derived both primitive endothelial cells and NPCs from human pluripotent stem cells. When these partially differentiating neural populations (expressing WNT7A/B) are cocultured with endothelial cells, hallmark BBB properties (e.g., TJs, Glut-1 expression, and efflux transporter functionality) become more pronounced [277]. In adult-like systems, astrocyte cocultures are essential for maintaining high TEER values and polarized transporter expression [278]; however, achieving in vitro resistance that matches in vivo levels (~6000 Ω × cm^2^ in some rodent models) remains challenging [279,280,281].

The ultimate advantage of stem cell-based BBB models is their adaptability to disease modeling. iPSCs derived from patients can be differentiated into neurons, astrocytes, pericytes, and BMECs, enabling the study of how specific genetic mutations (e.g., in familial AD) affect barrier integrity and disease progression [282,283,284]. Recent advancements emphasize the utility of TAT-functionalized DHAH-NLCs (transactivating transcriptional activator-functionalized docosahexaenoic acid-based nanostructured lipid carriers) as novel drug delivery platforms for neurodegenerative diseases. Using an in vitro BBB model derived from iPSCs, these nanostructured lipid carriers demonstrated successful BBB penetration, achieving a permeability of 0.4%. Furthermore, when loaded with glial cell-derived neurotrophic factor (GDNF), DHAH-NLCs activate the nuclear factor erythroid 2-related factor 2 (Nrf2) and heme oxygenase-1 (HO-1) pathways, enhancing the antioxidative response in microglia [285]. These findings underscore the critical role of iPSC-derived BBB models in evaluating targeted therapies and highlight the potential of combining stem cell-based systems with advanced nanotechnology to address neurodegenerative disorders.

**Figure 6:**
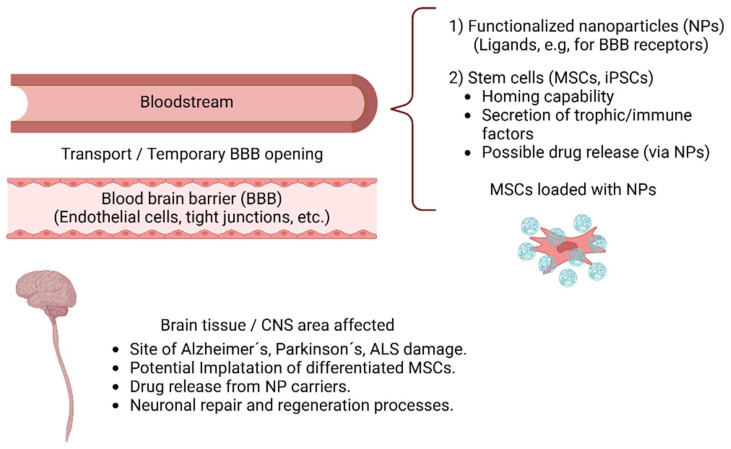
A combined nanoparticle and stem cell approach to bypass the blood–brain barrier in neurodegenerative diseases. Functionalized nanoparticles (NPs) can be administered to cross or temporarily open the BBB, carrying therapeutic molecules directly to affected brain regions. Mesenchymal stem cells (MSCs) or induced pluripotent stem cells (iPSCs) may also be loaded with NPs, taking advantage of their homing properties and the secretion of beneficial factors. Created in https://BioRender.com (accessed on 17 February 2025).

Continued refinement of these platforms promises to elucidate fundamental BBB mechanisms, accelerating the development of targeted therapies for neurodegenerative disorders. Taken together, these extensive findings illustrate how leveraging advanced nanotechnology, spanning polymeric NPs, liposomes, polymer micelles, and other innovative carriers, alongside stem cell-based approaches, can help overcome the formidable challenges posed by the BBB in neurodegenerative diseases (Figure 6).

## 9. Conclusions

NP-based therapies hold great promise for addressing the challenges posed by the BBB in treating neurodegenerative diseases. These technologies have shown the ability to enhance drug delivery and improve BBB integrity. When integrated with stem cell therapies, they can also support tissue regeneration and reduce neuro-inflammation. Nevertheless, obstacles remain, particularly regarding long-term safety, NP accumulation, and the need to optimize properties such as targeting accuracy and stability.

Moving forward, research must prioritize improving NP design, refining disease-specific models, and conducting robust clinical studies. These steps are essential to fully unlock the potential of NP therapies, ultimately enabling more effective treatments that can slow disease progression and help restore lost neurological functions.

## Figures and Tables

**Table 2:** Nanoparticle-based strategies to counteract neuro-inflammation and restore BBB integrity. Inflammatory mediators such as IL-1β and TNF-α are key drivers of BBB damage. NPs designed to neutralize these mediators or deliver genes that counteract their effects can preserve BBB integrity and promote a more favorable environment for neural repair.

Therapeutic Strategy	Mechanism of Action	Disease Model	Outcome	References
Blocking Endothelial Adhesion Molecules	Nanoparticles delivering siRNA/antibodies against VCAM-1 or ICAM-1	Multiple sclerosis, AD	Reduced leukocyte infiltration, decreased microglial activation	[184,210,211,213,214]
Delivering Anti-Inflammatory Drugs	Liposomes loaded with curcumin, NSAIDs, or antioxidants	AD, PD, ALS	Lowered pro-inflammatory cytokines, improved neuronal survival	[157,158,206,207,208,215]
Scavenging Reactive Oxygen Species	Metal oxide or polymeric NPs with ROS scavengers (e.g., catalase mimetics)	Models of vascular dementia	Restoration of tight junction expression, BBB protection	[96,198,199,200,201,202]
Gene Therapy (miRNA, siRNA, plasmids)	Inhibition of pro-inflammatory genes or induction of protective factors	AD, PD, ischemic stroke models	Suppressed inflammatory cascades, enhanced BBB function	[76,125,153]
Stem Cell-Derived Nanovesicles (Exosomes)	Modulate inflammation via paracrine signaling and microRNA transfer	AD, TBI, PD	Attenuated gliosis, improved neuronal regeneration	[161,216,217]

## Data Availability

Not applicable.

## References

[1] Huang Y., Li Y., Pan H., Han L. (2023). Global, Regional, and National Burden of Neurological Disorders in 204 Countries and Territories Worldwide. J. Glob. Health.

[2] Livingston G., Huntley J., Sommerlad A., Ames D., Ballard C., Banerjee S., Brayne C., Burns A., Cohen-Mansfield J., Cooper C. (2020). Dementia Prevention, Intervention, and Care: 2020 Report of the Lancet Commission. Lancet.

[3] Zahra W., Rai S.N., Birla H., Singh S.S., Dilnashin H., Rathore A.S., Singh S.P. (2020). The Global Economic Impact of Neurodegenerative Diseases: Opportunities and Challenges. Bioeconomy for Sustainable Development.

[4] Prince M., Ali G.C., Guerchet M., Prina A.M., Albanese E., Wu Y.T. (2016). Recent Global Trends in the Prevalence and Incidence of Dementia, and Survival with Dementia. Alzheimers Res. Ther..

[5] Etters L., Goodall D., Harrison B.E. (2008). Caregiver Burden among Dementia Patient Caregivers: A Review of the Literature. J. Am. Acad. Nurse Pract..

[6] Feigin V.L., Nichols E., Alam T., Bannick M.S., Beghi E., Blake N., Culpepper W.J., Dorsey E.R., Elbaz A., Ellenbogen R.G. (2019). Global, Regional, and National Burden of Neurological Disorders, 1990–2016: A Systematic Analysis for the Global Burden of Disease Study 2016. Lancet Neurol..

[7] Ou Z., Pan J., Tang S., Duan D., Yu D., Nong H., Wang Z. (2021). Global Trends in the Incidence, Prevalence, and Years Lived with Disability of Parkinson’s Disease in 204 Countries/Territories From 1990 to 2019. Front. Public Health.

[8] Boentert M., Hermann A., Großkreutz J. (2023). Amyotrophic Lateral Sclerosis: Advances and Prospects. J. Clin. Med..

[9] Bloom G.S. (2014). Amyloid-β and Tau: The Trigger and Bullet in Alzheimer Disease Pathogenesis. JAMA Neurol..

[10] Zhou Z.D., Yi L.X., Wang D.Q., Lim T.M., Tan E.K. (2023). Role of Dopamine in the Pathophysiology of Parkinson’s Disease. Transl. Neurodegener..

[11] Al-Khayri J.M., Ravindran M., Banadka A., Vandana C.D., Priya K., Nagella P., Kukkemane K. (2024). Amyotrophic Lateral Sclerosis: Insights and New Prospects in Disease Pathophysiology, Biomarkers and Therapies. Pharmaceuticals.

[12] Estudillo E., López-Ornelas A., Rodríguez-Oviedo A., de la Cruz N.G., Vargas-Hernández M.A., Jiménez A. (2023). Thinking Outside the Black Box: Are the Brain Endothelial Cells the New Main Target in Alzheimer’s Disease?. Neural Regen. Res..

[13] Persidsky Y., Ramirez S.H., Haorah J., Kanmogne G.D. (2006). Blood-Brain Barrier: Structural Components and Function under Physiologic and Pathologic Conditions. J. Neuroimmune Pharmacol..

[14] Knox E.G., Aburto M.R., Clarke G., Cryan J.F., O’Driscoll C.M. (2022). The Blood-Brain Barrier in Aging and Neurodegeneration. Mol. Psychiatry.

[15] Wisniewski H.M., Vorbrodt A.W., Wegiel J. (1997). Amyloid Angiopathy and Blood-Brain Barrier Changes in Alzheimer’s Disease. Ann. N. Y. Acad. Sci..

[16] Wu Y.C., Sonninen T.M., Peltonen S., Koistinaho J., Lehtonen Š. (2021). Blood–Brain Barrier and Neurodegenerative Diseases—Modeling with Ipsc-derived Brain Cells. Int. J. Mol. Sci..

[17] Pietronigro E., Zenaro E., Constantin G. (2016). Imaging of Leukocyte Trafficking in Alzheimer’s Disease. Front. Immunol..

[18] Wu J.R., Hernandez Y., Miyasaki K.F., Kwon E.J. (2023). Engineered Nanomaterials That Exploit Blood-Brain Barrier Dysfunction for Delivery to the Brain. Adv. Drug Deliv. Rev..

[19] Sweeney M.D., Sagare A.P., Zlokovic B.V. (2018). Blood–Brain Barrier Breakdown in Alzheimer Disease and Other Neurodegenerative Disorders. Nat. Rev. Neurol..

[20] Pan Y., Nicolazzo J.A. (2018). Impact of Aging, Alzheimer’s Disease and Parkinson’s Disease on the Blood-Brain Barrier Transport of Therapeutics. Adv. Drug Deliv. Rev..

[21] Kortekaas R., Leenders K.L., Van Oostrom J.C.H., Vaalburg W., Bart J., Willemsen A.T.M., Hendrikse N.H. (2005). Blood–Brain Barrier Dysfunction in Parkinsonian Midbrain in Vivo. Ann. Neurol..

[22] Rossa J., Ploeger C., Vorreiter F., Saleh T., Protze J., Günzel D., Wolburg H., Krause G., Piontek J. (2014). Claudin-3 and Claudin-5 Protein Folding and Assembly into the Tight Junction Are Controlled by Non-Conserved Residues in the Transmembrane 3 (TM3) and Extracellular Loop 2 (ECL2) Segments. J. Biol. Chem..

[23] Simpson I.A., Chundu K.R., Davies-Hill T., Honer W.G., Davies P. (1994). Decreased Concentrations of GLUT1 and GLUT3 Glucose Transporters in the Brains of Patients with Alzheimer’s Disease. Ann. Neurol..

[24] Wang Q., Huang X., Su Y., Yin G., Wang S., Yu B., Li H., Qi J., Chen H., Zeng W. (2022). Activation of Wnt/β-Catenin Pathway Mitigates Blood-Brain Barrier Dysfunction in Alzheimer’s Disease. Brain.

[25] Zenaro E., Piacentino G., Constantin G. (2017). The Blood-Brain Barrier in Alzheimer’s Disease. Neurobiol. Dis..

[26] Thomsen M.S., Routhe L.J., Moos T. (2017). The Vascular Basement Membrane in the Healthy and Pathological Brain. J. Cereb. Blood Flow. Metab..

[27] Archie S.R., Al Shoyaib A., Cucullo L. (2021). Blood-Brain Barrier Dysfunction in CNS Disorders and Putative Therapeutic Targets: An Overview. Pharmaceutics.

[28] D’Souza A., Dave K.M., Stetler R.A., S. Manickam D. (2021). Targeting the Blood-Brain Barrier for the Delivery of Stroke Therapies. Adv. Drug Deliv. Rev..

[29] Halliday M.R., Rege S.V., Ma Q., Zhao Z., Miller C.A., Winkler E.A., Zlokovic B.V. (2016). Accelerated Pericyte Degeneration and Blood-Brain Barrier Breakdown in Apolipoprotein E4 Carriers with Alzheimer’s Disease. J. Cereb. Blood Flow. Metab..

[30] Pisani V., Stefani A., Pierantozzi M., Natoli S., Stanzione P., Franciotta D., Pisani A. (2012). Increased Blood-Cerebrospinal Fluid Transfer of Albumin in Advanced Parkinson’s Disease. J. Neuroinflammation.

[31] Simon D.K., Tanner C.M., Brundin P. (2020). Parkinson Disease Epidemiology, Pathology, Genetics, and Pathophysiology. Clin. Geriatr. Med..

[32] Gray M.T., Woulfe J.M. (2015). Striatal Blood-Brain Barrier Permeability in Parkinson’s Disease. J. Cereb. Blood Flow. Metab..

[33] Pienaar I.S., Lee C.H., Elson J.L., McGuinness L., Gentleman S.M., Kalaria R.N., Dexter D.T. (2015). Deep-Brain Stimulation Associates with Improved Microvascular Integrity in the Subthalamic Nucleus in Parkinson’s Disease. Neurobiol. Dis..

[34] Lan G., Wang P., Chan R.B., Liu Z., Yu Z., Liu X., Yang Y., Zhang J. (2022). Astrocytic VEGFA: An Essential Mediator in Blood–Brain-Barrier Disruption in Parkinson’s Disease. Glia.

[35] Sweeney M.D., Zhao Z., Montagne A., Nelson A.R., Zlokovic B.V. (2019). Blood-Brain Barrier: From Physiology to Disease and Back. Physiol. Rev..

[36] Kurz C., Walker L., Rauchmann B.S., Perneczky R. (2022). Dysfunction of the Blood–Brain Barrier in Alzheimer’s Disease: Evidence from Human Studies. Neuropathol. Appl. Neurobiol..

[37] Głuchowska K., Pliszka M., Szablewski L. (2021). Expression of Glucose Transporters in Human Neurodegenerative Diseases. Biochem. Biophys. Res. Commun..

[38] Kadry H., Noorani B., Cucullo L. (2020). A Blood–Brain Barrier Overview on Structure, Function, Impairment, and Biomarkers of Integrity. Fluids Barriers CNS.

[39] Versele R., Sevin E., Gosselet F., Fenart L., Candela P. (2022). TNF-α and IL-1β Modulate Blood-Brain Barrier Permeability and Decrease Amyloid-β Peptide Efflux in a Human Blood-Brain Barrier Model. Int. J. Mol. Sci..

[40] Caserta M.T., Caccioppo D., Lapin G.D., Ragin A., Groothuis D.R. (1998). Blood-Brain Barrier Integrity in Alzheimer’s Disease Patients and Elderly Control Subjects. J. Neuropsychiatry Clin. Neurosci..

[41] Schlageter N.L., Carson R.E., Rapoport S.I. (1987). Examination of Blood—Brain Barrier Permeability in Dementia of the Alzheimer Type with [^68^Ga]EDTA and Positron Emission Tomography. J. Cereb. Blood Flow Metab..

[42] Starr J.M., Farrall A.J., Armitage P., McGurn B., Wardlaw J. (2009). Blood-Brain Barrier Permeability in Alzheimer’s Disease: A Case-Control MRI Study. Psychiatry Res. Neuroimaging.

[43] Bien-Ly N., Boswell C.A., Jeet S., Beach T.G., Hoyte K., Luk W., Shihadeh V., Ulufatu S., Foreman O., Lu Y. (2015). Lack of Widespread BBB Disruption in Alzheimer’s Disease Models: Focus on Therapeutic Antibodies. Neuron.

[44] Akhtar A., Andleeb A., Waris T.S., Bazzar M., Moradi A.R., Awan N.R., Yar M. (2021). Neurodegenerative Diseases and Effective Drug Delivery: A Review of Challenges and Novel Therapeutics. J. Control. Release.

[45] Pardridge W.M. (2020). Treatment of Alzheimer’s Disease and Blood–Brain Barrier Drug Delivery. Pharmaceuticals.

[46] Kuroiwa H., Yokoyama H., Kimoto H., Kato H., Araki T. (2010). Biochemical Alterations of the Striatum in an MPTP-Treated Mouse Model of Parkinson’s Disease. Metab. Brain Dis..

[47] Raza C., Anjum R. (2019). Parkinson’s Disease: Mechanisms, Translational Models and Management Strategies. Life Sci..

[48] Teleanu D.M., Chircov C., Grumezescu A.M., Teleanu R.I. (2019). Neurotoxicity of Nanomaterials: An up-to-Date Overview. Nanomaterials.

[49] Upadhyay R.K. (2014). Drug Delivery Systems, CNS Protection, and the Blood Brain Barrier. Biomed. Res. Int..

[50] Duan L., Li X., Ji R., Hao Z., Kong M., Wen X., Guan F., Ma S. (2023). Nanoparticle-Based Drug Delivery Systems: An Inspiring Therapeutic Strategy for Neurodegenerative Diseases. Polymers.

[51] Kingwell K. (2023). A New Shuttle for Drug Delivery across the Blood–Brain Barrier. Nat. Rev. Drug Discov..

[52] Pornnoppadol G., Bond L.G., Lucas M.J., Zupancic J.M., Kuo Y.H., Zhang B., Greineder C.F., Tessier P.M. (2024). Bispecific Antibody Shuttles Targeting CD98hc Mediate Efficient and Long-Lived Brain Delivery of IgGs. Cell Chem. Biol..

[53] Xie J., Gonzalez-Carter D., Tockary T.A., Nakamura N., Xue Y., Nakakido M., Akiba H., Dirisala A., Liu X., Toh K. (2020). Dual-Sensitive Nanomicelles Enhancing Systemic Delivery of Therapeutically Active Antibodies Specifically into the Brain. ACS Nano.

[54] Nair L.V., Nair R.V., Shenoy S.J., Thekkuveettil A., Jayasree R.S. (2017). Blood Brain Barrier Permeable Gold Nanocluster for Targeted Brain Imaging and Therapy: An: In Vitro and in Vivo Study. J. Mater. Chem. B.

[55] Vargas-Barona A., Bernáldez-Sarabia J., Castro-Ceseña A.B. (2024). Lipid-Polymer Hybrid Nanoparticles Loaded with N-Acetylcysteine for the Modulation of Neuroinflammatory Biomarkers in Human IPSC-Derived PSEN2 (N141I) Astrocytes as a Model of Alzheimer’s Disease. J. Mater. Chem. B.

[56] Yu Y.J., Zhang Y., Kenrick M., Hoyte K., Luk W., Lu Y., Atwal J., Elliott J.M., Prabhu S., Watts R.J. (2011). Boosting Brain Uptake of a Therapeutic Antibody by Reducing Its Affinity for a Transcytosis Target. Sci. Transl. Med..

[57] Mohammadpour R., Dobrovolskaia M.A., Cheney D.L., Greish K.F., Ghandehari H. (2019). Subchronic and Chronic Toxicity Evaluation of Inorganic Nanoparticles for Delivery Applications. Adv. Drug Deliv. Rev..

[58] Ray S., Sinha P., Laha B., Maiti S., Bhattacharyya U.K., Nayak A.K. (2018). Polysorbate 80 Coated Crosslinked Chitosan Nanoparticles of Ropinirole Hydrochloride for Brain Targeting. J. Drug Deliv. Sci. Technol..

[59] Zhang W., Mehta A., Tong Z., Esser L., Voelcker N.H., Zhang W., Mehta A., Tong Z., Esser L., Voelcker N.H. (2021). Development of Polymeric Nanoparticles for Blood–Brain Barrier Transfer—Strategies and Challenges. Adv. Sci..

[60] Jose S., Sowmya S., Cinu T.A., Aleykutty N.A., Thomas S., Souto E.B. (2014). Surface Modified PLGA Nanoparticles for Brain Targeting of Bacoside-A. Eur. J. Pharm. Sci..

[61] Monsalve Y., Tosi G., Ruozi B., Belletti D., Vilella A., Zoli M., Vandelli M.A., Forni F., López B.L., Sierra L. (2015). PEG-g-Chitosan Nanoparticles Functionalized with the Monoclonal Antibody OX26 for Brain Drug Targeting. Nanomedicine.

[62] Yu S., Xu X., Feng J., Liu M., Hu K. (2019). Chitosan and Chitosan Coating Nanoparticles for the Treatment of Brain Disease. Int. J. Pharm..

[63] Joudeh N., Linke D. (2022). Nanoparticle Classification, Physicochemical Properties, Characterization, and Applications: A Comprehensive Review for Biologists. J. Nanobiotechnol..

[64] Toader C., Dumitru A.V., Eva L., Serban M., Covache-Busuioc R.A., Ciurea A.V. (2024). Nanoparticle Strategies for Treating CNS Disorders: A Comprehensive Review of Drug Delivery and Theranostic Applications. Int. J. Mol. Sci..

[65] Johnsen K.B., Bak M., Kempen P.J., Melander F., Burkhart A., Thomsen M.S., Nielsen M.S., Moos T., Andresen T.L. (2018). Antibody Affinity and Valency Impact Brain Uptake of Transferrin Receptor-Targeted Gold Nanoparticles. Theranostics.

[66] Guo S., Yi C.X. (2023). Cell Type-Targeting Nanoparticles in Treating Central Nervous System Diseases: Challenges and Hopes. Nanotechnol. Rev..

[67] Asimakidou E., Tan J.K.S., Zeng J., Lo C.H. (2024). Blood–Brain Barrier-Targeting Nanoparticles: Biomaterial Properties and Biomedical Applications in Translational Neuroscience. Pharmaceuticals.

[68] Ceña V., Játiva P. (2018). Nanoparticle Crossing of Blood–Brain Barrier: A Road to New Therapeutic Approaches to Central Nervous System Diseases. Nanomedicine.

[69] De Jong W.H., Hagens W.I., Krystek P., Burger M.C., Sips A.J.A.M., Geertsma R.E. (2008). Particle Size-Dependent Organ Distribution of Gold Nanoparticles after Intravenous Administration. Biomaterials.

[70] Hashemi-Moghaddam H., Kazemi-Bagsangani S., Jamili M., Zavareh S. (2016). Evaluation of Magnetic Nanoparticles Coated by 5-Fluorouracil Imprinted Polymer for Controlled Drug Delivery in Mouse Breast Cancer Model. Int. J. Pharm..

[71] Perumal S. (2022). Polymer Nanoparticles: Synthesis and Applications. Polymers.

[72] Kumar S., Maiti P. (2016). Controlled Biodegradation of Polymers Using Nanoparticles and Its Application. RSC Adv..

[73] Vodyashkin A.A., Kezimana P., Vetcher A.A., Stanishevskiy Y.M. (2022). Biopolymeric Nanoparticles–Multifunctional Materials of the Future. Polymers.

[74] Wang Y., Qin B., Xia G., Choi S.H. (2021). FDA’s Poly (Lactic-Co-Glycolic Acid) Research Program and Regulatory Outcomes. AAPS J..

[75] Hu K., Li J., Shen Y., Lu W., Gao X., Zhang Q., Jiang X. (2009). Lactoferrin-Conjugated PEG-PLA Nanoparticles with Improved Brain Delivery: In Vitro and in Vivo Evaluations. J. Control Release.

[76] Li W., Qiu J., Li X.L., Aday S., Zhang J., Conley G., Xu J., Joseph J., Lan H., Langer R. (2021). BBB Pathophysiology–Independent Delivery of SiRNA in Traumatic Brain Injury. Sci. Adv..

[77] Katila N., Duwa R., Bhurtel S., Khanal S., Maharjan S., Jeong J.H., Lee S., Choi D.Y., Yook S. (2022). Enhancement of Blood–Brain Barrier Penetration and the Neuroprotective Effect of Resveratrol. J. Control. Release.

[78] Bhardwaj H., Jangde R.K. (2023). Current Updated Review on Preparation of Polymeric Nanoparticles for Drug Delivery and Biomedical Applications. Next Nanotechnol..

[79] Akbarzadeh A., Rezaei-Sadabady R., Davaran S., Joo S.W., Zarghami N., Hanifehpour Y., Samiei M., Kouhi M., Nejati-Koshki K. (2013). Liposome: Classification, Preparation, and Applications. Nanoscale Res. Lett..

[80] Nsairat H., Khater D., Sayed U., Odeh F., Al Bawab A., Alshaer W. (2022). Liposomes: Structure, Composition, Types, and Clinical Applications. Heliyon.

[81] Torchilinl V.P., Papisov M.I. (1994). Why Do Polyethylene Glycol-Coated Liposomes Circulate so Long?: Molecular Mechanism of Liposome Steric Protection with Polyethylene Glycol: Role of Polymer Chain Flexibility. J. Liposome Res..

[82] Barenholz Y. (2012). Doxil^®^—The First FDA-Approved Nano-Drug: Lessons Learned. J. Control. Release.

[83] Senapati S., Tripathi K., Awad K., Rahimipour S. (2024). Multifunctional Liposomes Targeting Amyloid-β Oligomers for Early Diagnosis and Therapy of Alzheimer’s Disease. Small.

[84] Chen Z., Kankala R.K., Long L., Xie S., Chen A., Zou L. (2023). Current Understanding of Passive and Active Targeting Nanomedicines to Enhance Tumor Accumulation. Coord. Chem. Rev..

[85] Akinc A., Maier M.A., Manoharan M., Fitzgerald K., Jayaraman M., Barros S., Ansell S., Du X., Hope M.J., Madden T.D. (2019). The Onpattro Story and the Clinical Translation of Nanomedicines Containing Nucleic Acid-Based Drugs. Nature Nanotechnol..

[86] Borys N., Dewhirst M.W. (2021). Drug Development of Lyso-Thermosensitive Liposomal Doxorubicin: Combining Hyperthermia and Thermosensitive Drug Delivery. Adv. Drug Deliv. Rev..

[87] Gong X., Fan X., He Y., Wang Y., Zhou F., Yang B. (2022). A PH-Sensitive Liposomal Co-Delivery of Fingolimod and Ammonia Borane for Treatment of Intracerebral Hemorrhage. Nanophotonics.

[88] Ghasemiyeh P., Mohammadi-Samani S. (2018). Solid Lipid Nanoparticles and Nanostructured Lipid Carriers as Novel Drug Delivery Systems: Applications, Advantages and Disadvantages. Res. Pharm. Sci..

[89] Haider M.S., Lübtow M.M., Endres S., Forster S., Flegler V.J., Böttcher B., Aseyev V., Pöppler A.C., Luxenhofer R., Luxenhofer R. (2020). Think Beyond the Core: Impact of the Hydrophilic Corona on Drug Solubilization Using Polymer Micelles. ACS Appl. Mater. Interfaces.

[90] Hwang D., Ramsey J.D., Kabanov A.V. (2020). Polymeric Micelles for the Delivery of Poorly Soluble Drugs: From Nanoformulation to Clinical Approval. Adv. Drug Deliv. Rev..

[91] Dattani S., Li X., Lampa C., Lechuga-Ballesteros D., Barriscale A., Damadzadeh B., Jasti B.R. (2023). A Comparative Study on Micelles, Liposomes and Solid Lipid Nanoparticles for Paclitaxel Delivery. Int. J. Pharm..

[92] Itagaki M., Nasu Y., Sugiyama C., Nakase I., Kamei N. (2023). A Universal Method to Analyze Cellular Internalization Mechanisms via Endocytosis without Non-Specific Cross-Effects. FASEB J..

[93] Bareford L.M., Swaan P.W. (2007). Endocytic Mechanisms for Targeted Drug Delivery. Adv. Drug Deliv. Rev..

[94] Lu Y., Zhang E., Yang J., Cao Z. (2018). Strategies to Improve Micelle Stability for Drug Delivery. Nano Res..

[95] Ouyang N., Yang C., Li X., Zheng Z., Xu Y., Wang Y., Xiong W., Wu H. (2024). Development of Lactoferrin-Coated Multifunctional Copolymer Micelles to Cross the Blood-Brain Barrier. Drug Deliv. Transl. Res..

[96] You Y., Liu Y., Ma C., Xu J., Xie L., Tong S., Sun Y., Ma F., Huang Y., Liu J. (2023). Surface-Tethered ROS-Responsive Micelle Backpacks for Boosting Mesenchymal Stem Cell Vitality and Modulating Inflammation in Ischemic Stroke Treatment. J. Control. Release.

[97] Ishii T., Asai T., Oyama D., Fukuta T., Yasuda N., Shimizu K., Minamino T., Oku N. (2012). Amelioration of Cerebral Ischemia-Reperfusion Injury Based on Liposomal Drug Delivery System with Asialo-Erythropoietin. J. Control Release.

[98] Wilhelm I., Nyúl-Tóth Á., Suciu M., Hermenean A., Krizbai I.A. (2016). Heterogeneity of the Blood-Brain Barrier. Tissue Barriers.

[99] Sheikov N., McDannold N., Sharma S., Hynynen K. (2008). Effect of Focused Ultrasound Applied with an Ultrasound Contrast Agent on the Tight Junctional Integrity of the Brain Microvascular Endothelium. Ultrasound Med. Biol..

[100] Chen H., Konofagou E.E. (2014). The Size of Blood-Brain Barrier Opening Induced by Focused Ultrasound Is Dictated by the Acoustic Pressure. J. Cereb. Blood Flow. Metab..

[101] Worzfeld T., Schwaninger M. (2016). Apicobasal Polarity of Brain Endothelial Cells. J. Cereb. Blood Flow. Metab..

[102] Profaci C.P., Munji R.N., Pulido R.S., Daneman R. (2020). The Blood-Brain Barrier in Health and Disease: Important Unanswered Questions. J. Exp. Med..

[103] Giugliani R., Giugliani L., De Oliveira Poswar F., Donis K.C., Corte A.D., Schmidt M., Boado R.J., Nestrasil I., Nguyen C., Chen S. (2018). Neurocognitive and Somatic Stabilization in Pediatric Patients with Severe Mucopolysaccharidosis Type I after 52 Weeks of Intravenous Brain-Penetrating Insulin Receptor Antibody-Iduronidase Fusion Protein (Valanafusp Alpha): An Open Label Phase 1-2 Trial. Orphanet J. Rare Dis..

[104] Pulgar V.M. (2019). Transcytosis to Cross the Blood Brain Barrier, New Advancements and Challenges. Front. Neurosci..

[105] Hervé F., Ghinea N., Scherrmann J.M. (2008). CNS Delivery via Adsorptive Transcytosis. AAPS J..

[106] Haqqani A.S., Bélanger K., Stanimirovic D.B. (2024). Receptor-Mediated Transcytosis for Brain Delivery of Therapeutics: Receptor Classes and Criteria. Front. Drug Deliv..

[107] Yang A.C., Stevens M.Y., Chen M.B., Lee D.P., Stähli D., Gate D., Contrepois K., Chen W., Iram T., Zhang L. (2020). Physiological Blood-Brain Transport Is Impaired with Age by a Shift in Transcytosis. Nature.

[108] Yang A.C., Vest R.T., Kern F., Lee D.P., Agam M., Maat C.A., Losada P.M., Chen M.B., Schaum N., Khoury N. (2022). A Human Brain Vascular Atlas Reveals Diverse Mediators of Alzheimer’s Risk. Nature.

[109] Juul Rasmussen I., Tybjærg-Hansen A., Rasmussen K.L., Nordestgaard B.G., Frikke-Schmidt R. (2019). Blood-Brain Barrier Transcytosis Genes, Risk of Dementia and Stroke: A Prospective Cohort Study of 74,754 Individuals. Eur. J. Epidemiol..

[110] Hou X., Zaks T., Langer R., Dong Y. (2021). Lipid Nanoparticles for MRNA Delivery. Nat. Rev. Mater..

[111] Jain D., Hasan N., Zafar S., Thakur J., Haider K., Parvez S., Ahmad F.J. (2023). Transferrin Functionalized Nanostructured Lipid Carriers for Targeting Rivastigmine and Resveratrol to Alzheimer’s Disease: Synthesis, in Vitro Characterization and Brain Uptake Analysis. J. Drug Deliv. Sci. Technol..

[112] McClements D.J. (2018). Encapsulation, Protection, and Delivery of Bioactive Proteins and Peptides Using Nanoparticle and Microparticle Systems: A Review. Adv. Colloid. Interface Sci..

[113] Jeong S.M., Shin D.W., Yoo T.G., Cho M.H., Jang W., Lee J., Kim S.Y. (2021). Association between Statin Use and Alzheimer’s Disease with Dose Response Relationship. Sci. Rep..

[114] Feron O., Dessy C., Desager J.P., Balligand J.L. (2001). Hydroxy-Methylglutaryl-Coenzyme A Reductase Inhibition Promotes Endothelial Nitric Oxide Synthase Activation through a Decrease in Caveolin Abundance. Circulation.

[115] Fessel J. (2020). Caveolae, CD109, and Endothelial Cells as Targets for Treating Alzheimer’s Disease. Alzheimers Dement..

[116] Mishra V., Mahor S., Rawat A., Gupta P.N., Dubey P., Khatri K., Vyas S.P. (2006). Targeted Brain Delivery of AZT via Transferrin Anchored Pegylated Albumin Nanoparticles. J. Drug Target..

[117] Wiley D.T., Webster P., Gale A., Davis M.E. (2013). Transcytosis and Brain Uptake of Transferrin-Containing Nanoparticles by Tuning Avidity to Transferrin Receptor. Proc. Natl. Acad. Sci. USA.

[118] Ulbrich K., Knobloch T., Kreuter J. (2011). Targeting the Insulin Receptor: Nanoparticles for Drug Delivery across the Blood-Brain Barrier (BBB). J. Drug Target..

[119] Kaya S., Callan B., Hawthorne S. (2023). Non-Invasive, Targeted Nanoparticle-Mediated Drug Delivery across a Novel Human BBB Model. Pharmaceutics.

[120] Liu Z., Gao X., Kang T., Jiang M., Miao D., Gu G., Hu Q., Song Q., Yao L., Tu Y. (2013). B6 Peptide-Modified PEG-PLA Nanoparticles for Enhanced Brain Delivery of Neuroprotective Peptide. Bioconjug. Chem..

[121] Georgieva J.V., Brinkhuis R.P., Stojanov K., Weijers C.A.G.M., Zuilhof H., Rutjes F.P.J.T., Hoekstra D., Van Hest J.C.M., Zuhorn I.S. (2012). Peptide-Mediated Blood-Brain Barrier Transport of Polymersomes. Angew. Chem. Int. Ed. Engl..

[122] Yin T., Yang L., Liu Y., Zhou X., Sun J., Liu J. (2015). Sialic Acid (SA)-Modified Selenium Nanoparticles Coated with a High Blood-Brain Barrier Permeability Peptide-B6 Peptide for Potential Use in Alzheimer’s Disease. Acta Biomater..

[123] Li Y.H., Chen Z.X., Lu Z.G., Yang Q.H., Liu L.Y., Jiang Z.T., Zhang L.Q., Zhang X., Qing H. (2018). “Cell-Addictive” Dual-Target Traceable Nanodrug for Parkinson’s Disease Treatment via Flotillins Pathway. Theranostics.

[124] Novorolsky R.J., Kasheke G.D.S., Hakim A., Foldvari M., Dorighello G.G., Sekler I., Vuligonda V., Sanders M.E., Renden R.B., Wilson J.J. (2023). Preserving and Enhancing Mitochondrial Function after Stroke to Protect and Repair the Neurovascular Unit: Novel Opportunities for Nanoparticle-Based Drug Delivery. Front. Cell Neurosci..

[125] Wang P., Zheng X., Guo Q., Yang P., Pang X., Qian K., Lu W., Zhang Q., Jiang X. (2018). Systemic Delivery of BACE1 SiRNA through Neuron-Targeted Nanocomplexes for Treatment of Alzheimer’s Disease. J. Control Release.

[126] Rotman M., Welling M.M., Bunschoten A., De Backer M.E., Rip J., Nabuurs R.J.A., Gaillard P.J., Van Buchem M.A., Van Der Maarel S.M., Van Der Weerd L. (2015). Enhanced Glutathione PEGylated Liposomal Brain Delivery of an Anti-Amyloid Single Domain Antibody Fragment in a Mouse Model for Alzheimer’s Disease. J. Control Release.

[127] Yang H., Mu W., Wei D., Zhang Y., Duan Y., Gao J., Gong X., Wang H., Wu X., Tao H. (2020). A Novel Targeted and High-Efficiency Nanosystem for Combinational Therapy for Alzheimer’s Disease. Adv. Sci..

[128] Huang R., Han L., Li J., Ren F., Ke W., Jiang C., Pei Y. (2009). Neuroprotection in a 6-Hydroxydopamine-Lesioned Parkinson Model Using Lactoferrin-Modified Nanoparticles. J. Gene Med..

[129] Guérit S., Fidan E., Macas J., Czupalla C.J., Figueiredo R., Vijikumar A., Yalcin B.H., Thom S., Winter P., Gerhardt H. (2021). Astrocyte-Derived Wnt Growth Factors Are Required for Endothelial Blood-Brain Barrier Maintenance. Prog. Neurobiol..

[130] Alvarez J.I., Dodelet-Devillers A., Kebir H., Ifergan I., Fabre P.J., Terouz S., Sabbagh M., Wosik K., Bourbonnière L., Bernard M. (2011). The Hedgehog Pathway Promotes Blood-Brain Barrier Integrity and CNS Immune Quiescence. Science.

[131] Augustin H.G., Young Koh G., Thurston G., Alitalo K. (2009). Control of Vascular Morphogenesis and Homeostasis through the Angiopoietin–Tie System. Nat. Rev. Mol. Cell Biol..

[132] Nourhaghighi N., Teichert-Kuliszewska K., Davis J., Stewart D.J., Nag S. (2003). Altered Expression of Angiopoietins during Blood-Brain Barrier Breakdown and Angiogenesis. Lab. Investig..

[133] Surnar B., Basu U., Banik B., Ahmad A., Marples B., Kolishetti N., Dhar S. (2018). Nanotechnology-Mediated Crossing of Two Impermeable Membranes to Modulate the Stars of the Neurovascular Unit for Neuroprotection. Proc. Natl. Acad. Sci. USA.

[134] Gromnicova R., Davies H.A., Sreekanthreddy P., Romero I.A., Lund T., Roitt I.M., Phillips J.B., Male D.K. (2013). Glucose-Coated Gold Nanoparticles Transfer across Human Brain Endothelium and Enter Astrocytes in Vitro. PLoS ONE.

[135] Guda M.R., Labak C.M., Omar S.I., Asuthkar S., Airala S., Tuszynski J., Tsung A.J., Velpula K.K. (2019). GLUT1 and TUBB4 in Glioblastoma Could Be Efficacious Targets. Cancers.

[136] Boado R.J., Black K.L., Pardridge W.M. (1994). Gene Expression of GLUT3 and GLUT1 Glucose Transporters in Human Brain Tumors. Brain Res. Mol. Brain Res..

[137] Tanvir A., Jo J., Park S.M. (2024). Targeting Glucose Metabolism: A Novel Therapeutic Approach for Parkinson’s Disease. Cells.

[138] Kyrtata N., Emsley H.C.A., Sparasci O., Parkes L.M., Dickie B.R. (2021). A Systematic Review of Glucose Transport Alterations in Alzheimer’s Disease. Front. Neurosci..

[139] Gamberino W.C., Brennan W.A. (1994). Glucose Transporter Isoform Expression in Huntington’s Disease Brain. J. Neurochem..

[140] Zhao Y., Gan L., Ren L., Lin Y., Ma C., Lin X. (2022). Factors Influencing the Blood-Brain Barrier Permeability. Brain Res..

[141] Berzin T.M., Zipser B.D., Rafii M.S., Kuo-Leblanc V., Yancopouloš G.D., Glass D.J., Fallon J.R., Stopa E.G. (2000). Agrin and Microvascular Damage in Alzheimer’s Disease. Neurobiol. Aging.

[142] Donahue J.E., Berzin T.M., Rafii M.S., Glass D.J., Yancopoulos G.D., Fallon J.R., Stopa E.G. (1999). Agrin in Alzheimer’s Disease: Altered Solubility and Abnormal Distribution within Microvasculature and Brain Parenchyma. Proc. Natl. Acad. Sci. USA.

[143] Kumarasamy M., Sosnik A. (2021). Heterocellular Spheroids of the Neurovascular Blood-Brain Barrier as a Platform for Personalized Nanoneuromedicine. iScience.

[144] Vashist A., Manickam P., Raymond A.D., Arias A.Y., Kolishetti N., Vashist A., Arias E., Nair M. (2023). Recent Advances in Nanotherapeutics for Neurological Disorders. ACS Appl. Bio Mater..

[145] Pondman K., Le Gac S., Kishore U. (2023). Nanoparticle-Induced Immune Response: Health Risk versus Treatment Opportunity?. Immunobiology.

[146] Aljabali A.A., Obeid M.A., Bashatwah R.M., Serrano-Aroca Á., Mishra V., Mishra Y., El-Tanani M., Hromić-Jahjefendić A., Kapoor D.N., Goyal R. (2023). Nanomaterials and Their Impact on the Immune System. Int. J. Mol. Sci..

[147] Xuan L., Ju Z., Skonieczna M., Zhou P.K., Huang R. (2023). Nanoparticles-Induced Potential Toxicity on Human Health: Applications, Toxicity Mechanisms, and Evaluation Models. MedComm.

[148] Shahalaei M., Azad A.K., Sulaiman W.M.A.W., Derakhshani A., Mofakham E.B., Mallandrich M., Kumarasamy V., Subramaniyan V. (2024). A Review of Metallic Nanoparticles: Present Issues and Prospects Focused on the Preparation Methods, Characterization Techniques, and Their Theranostic Applications. Front. Chem..

[149] Mitchell M.J., Billingsley M.M., Haley R.M., Wechsler M.E., Peppas N.A., Langer R. (2020). Engineering Precision Nanoparticles for Drug Delivery. Nat. Rev. Drug Discov..

[150] Hoshyar N., Gray S., Han H., Bao G. (2016). The Effect of Nanoparticle Size on in Vivo Pharmacokinetics and Cellular Interaction. Nanomedicine.

[151] Nayab D.E., Din F., Ali H., Kausar W.A., Urooj S., Zafar M., Khan I., Shabbir K., Khan G.M. (2023). Nano Biomaterials Based Strategies for Enhanced Brain Targeting in the Treatment of Neurodegenerative Diseases: An up-to-Date Perspective. J. Nanobiotechnology.

[152] Kumarasamy R.V., Natarajan P.M., Umapathy V.R., Roy J.R., Mironescu M., Palanisamy C.P. (2024). Clinical Applications and Therapeutic Potentials of Advanced Nanoparticles: A Comprehensive Review on Completed Human Clinical Trials. Front. Nanotechnol..

[153] Zhou Y., Zhu F., Liu Y., Zheng M., Wang Y., Zhang D., Anraku Y., Zou Y., Li J., Wu H. (2020). Blood-Brain Barrier–Penetrating SiRNA Nanomedicine for Alzheimer’s Disease Therapy. Sci. Adv..

[154] Bhattamisra S.K., Shak A.T., Xi L.W., Safian N.H., Choudhury H., Lim W.M., Shahzad N., Alhakamy N.A., Anwer M.K., Radhakrishnan A.K. (2020). Nose to Brain Delivery of Rotigotine Loaded Chitosan Nanoparticles in Human SH-SY5Y Neuroblastoma Cells and Animal Model of Parkinson’s Disease. Int. J. Pharm..

[155] Esteves M., Cristóvão A.C., Saraiva T., Rocha S.M., Baltazar G., Ferreira L., Bernardino L. (2015). Retinoic Acid-Loaded Polymeric Nanoparticles Induce Neuroprotection in a Mouse Model for Parkinson’s Disease. Front. Aging Neurosci..

[156] Tanifum E.A., Dasgupta I., Srivastava M., Bhavane R.C., Sun L., Berridge J., Pourgarzham H., Kamath R., Espinosa G., Cook S.C. (2012). Intravenous Delivery of Targeted Liposomes to Amyloid-β Pathology in APP/PSEN1 Transgenic Mice. PLoS ONE.

[157] Chen J., Dai W.T., He Z.M., Gao L., Huang X., Gong J.M., Xing H.Y., Chen W.D. (2013). Fabrication and Evaluation of Curcumin-Loaded Nanoparticles Based on Solid Lipid as a New Type of Colloidal Drug Delivery System. Indian. J. Pharm. Sci..

[158] Ganesan P., Kim B., Ramalaingam P., Karthivashan G., Revuri V., Park S., Kim J.S., Ko Y.T., Choi D.K. (2019). Antineuroinflammatory Activities and Neurotoxicological Assessment of Curcumin Loaded Solid Lipid Nanoparticles on LPS-Stimulated BV-2 Microglia Cell Models. Molecules.

[159] Song M., Sun Y., Luo Y., Zhu Y., Liu Y., Li H. (2018). Exploring the Mechanism of Inhibition of Au Nanoparticles on the Aggregation of Amyloid-β(16-22) Peptides at the Atom Level by All-Atom Molecular Dynamics. Int. J. Mol. Sci..

[160] Anand B.G., Wu Q., Karthivashan G., Shejale K.P., Amidian S., Wille H., Kar S. (2021). Mimosine Functionalized Gold Nanoparticles (Mimo-AuNPs) Suppress β-Amyloid Aggregation and Neuronal Toxicity. Bioact. Mater..

[161] Perets N., Betzer O., Shapira R., Brenstein S., Angel A., Sadan T., Ashery U., Popovtzer R., Offen D. (2019). Golden Exosomes Selectively Target Brain Pathologies in Neurodegenerative and Neurodevelopmental Disorders. Nano Lett..

[162] Muller A.P., Ferreira G.K., Pires A.J., de Bem Silveira G., de Souza D.L., de Brandolfi J.A., de Souza C.T., Paula M.M.S., Silveira P.C.L. (2017). Gold Nanoparticles Prevent Cognitive Deficits, Oxidative Stress and Inflammation in a Rat Model of Sporadic Dementia of Alzheimer’s Type. Mater. Sci. Eng. C.

[163] Praça C., Rai A., Santos T., Cristovão A.C., Pinho S.L., Cecchelli R., Dehouck M.P., Bernardino L., Ferreira L.S. (2018). A Nanoformulation for the Preferential Accumulation in Adult Neurogenic Niches. J. Control Release.

[164] Ahn H.S., Hwang J.Y., Kim M.S., Lee J.Y., Kim J.W., Kim H.S., Shin U.S., Knowles J.C., Kim H.W., Hyun J.K. (2015). Carbon-Nanotube-Interfaced Glass Fiber Scaffold for Regeneration of Transected Sciatic Nerve. Acta Biomater..

[165] Asefy Z., Hoseinnejhad S., Ceferov Z. (2021). Nanoparticles Approaches in Neurodegenerative Diseases Diagnosis and Treatment. Neurol. Sci..

[166] Fiorillo C., Bouitbir J., Basel U., Arpad Tosaki S., Wu G.-D., Wang L. (2021). Graphene Oxide and Reduced Graphene Oxide Exhibit Cardiotoxicity Through the Regulation of Lipid Peroxidation, Oxidative Stress, and Mitochondrial Dysfunction. Front. Cell Dev. Biol..

[167] Ren C., Hu X., Zhou Q., Ren C., Hu X., Zhou Q. (2018). Graphene Oxide Quantum Dots Reduce Oxidative Stress and Inhibit Neurotoxicity In Vitro and In Vivo through Catalase-Like Activity and Metabolic Regulation. Adv. Sci..

[168] Ramirez-Velez I., Belardi B. (2023). Storming the Gate: New Approaches for Targeting the Dynamic Tight Junction for Improved Drug Delivery. Adv. Drug Deliv. Rev..

[169] Abdullahi W., Tripathi D., Ronaldson P.T. (2018). Blood-Brain Barrier Dysfunction in Ischemic Stroke: Targeting Tight Junctions and Transporters for Vascular Protection. Am. J. Physiol. Cell Physiol..

[170] Anthony D.P., Hegde M., Shetty S.S., Rafic T., Mutalik S., Rao B.S.S. (2021). Targeting Receptor-Ligand Chemistry for Drug Delivery across Blood-Brain Barrier in Brain Diseases. Life Sci..

[171] Brunner J., Ragupathy S., Borchard G. (2021). Target Specific Tight Junction Modulators. Adv. Drug Deliv. Rev..

[172] Li C.H., Shyu M.K., Jhan C., Cheng Y.W., Tsai C.H., Liu C.W., Lee C.C., Chen R.M., Kang J.J. (2015). Gold Nanoparticles Increase Endothelial Paracellular Permeability by Altering Components of Endothelial Tight Junctions, and Increase Blood-Brain Barrier Permeability in Mice. Toxicol. Sci..

[173] González-Mariscal L., Posadas Y., Miranda J., Uc P., Ortega-Olvera J., Hernández S. (2016). Strategies That Target Tight Junctions for Enhanced Drug Delivery. Curr. Pharm. Des..

[174] Pinzon-Daza M., Campia I., Kopecka J., Garzon R., Ghigo D., Rigant C. (2013). Nanoparticle- and Liposome-Carried Drugs: New Strategies for Active Targeting and Drug Delivery across Blood-Brain Barrier. Curr. Drug Metab..

[175] Wen L., Tan Y., Dai S., Zhu Y., Meng T., Yang X., Liu Y., Liu X., Yuan H., Hu F. (2017). VEGF-Mediated Tight Junctions Pathological Fenestration Enhances Doxorubicin-Loaded Glycolipid-like Nanoparticles Traversing BBB for Glioblastoma-Targeting Therapy. Drug Deliv..

[176] Sasson E., Anzi S., Bell B., Yakovian O., Zorsky M., Deutsch U., Engelhardt B., Sherman E., Vatine G.D., Dzikowski R. (2021). Nano-Scale Architecture of Blood-Brain Barrier Tight-Junctions. Elife.

[177] Kim S., Moon G.J., Kim H.J., Kim D.G., Kim J., Nam Y., Sharma C., Leem E., Lee S., Kim K.S. (2022). Control of Hippocampal Prothrombin Kringle-2 (PKr-2) Expression Reduces Neurotoxic Symptoms in Five Familial Alzheimer’s Disease Mice. Br. J. Pharmacol..

[178] Lo Y.L., Lin H.C., Hong S.T., Chang C.H., Wang C.S., Lin A.M.Y. (2021). Lipid Polymeric Nanoparticles Modified with Tight Junction-Modulating Peptides Promote Afatinib Delivery across a Blood–Brain Barrier Model. Cancer Nanotechnol..

[179] Chen L., Deng H., Cui H., Fang J., Zuo Z., Deng J., Li Y., Wang X., Zhao L. (2017). Inflammatory Responses and Inflammation-Associated Diseases in Organs. Oncotarget.

[180] Prinz M., Priller J. (2014). Microglia and Brain Macrophages in the Molecular Age: From Origin to Neuropsychiatric Disease. Nat. Rev. Neurosci..

[181] Li Y., Du X.F., Liu C.S., Wen Z.L., Du J.L. (2012). Reciprocal Regulation between Resting Microglial Dynamics and Neuronal Activity in Vivo. Dev. Cell.

[182] Abbott N.J., Rönnbäck L., Hansson E. (2006). Astrocyte-Endothelial Interactions at the Blood-Brain Barrier. Nat. Rev. Neurosci..

[183] Liu R., Yang J., Liu L., Lu Z., Shi Z., Ji W., Shen J., Zhang X. (2019). An “Amyloid-β Cleaner” for the Treatment of Alzheimer’s Disease by Normalizing Microglial Dysfunction. Adv. Sci..

[184] Marcos-Contreras O.A., Greineder C.F., Kiseleva R.Y., Parhiz H., Walsh L.R., Zuluaga-Ramirez V., Myerson J.W., Hood E.D., Villa C.H., Tombacz I. (2020). Selective Targeting of Nanomedicine to Inflamed Cerebral Vasculature to Enhance the Blood–Brain Barrier. Proc. Natl. Acad. Sci. USA.

[185] Hess D.C., Thompson Y., Sprinkle A., Carroll J., Smith J. (1996). E-Selectin Expression on Human Brain Microvascular Endothelial Cells. Neurosci. Lett..

[186] Gholizadeh S., Visweswaran G.R.R., Storm G., Hennink W.E., Kamps J.A.A.M., Kok R.J. (2018). E-Selectin Targeted Immunoliposomes for Rapamycin Delivery to Activated Endothelial Cells. Int. J. Pharm..

[187] De Miguel Z., Khoury N., Betley M.J., Lehallier B., Willoughby D., Olsson N., Yang A.C., Hahn O., Lu N., Vest R.T. (2021). Exercise Plasma Boosts Memory and Dampens Brain Inflammation via Clusterin. Nature.

[188] Vainchtein I.D., Molofsky A.V. (2020). Astrocytes and Microglia: In Sickness and in Health. Trends Neurosci..

[189] Liddelow S.A., Marsh S.E., Stevens B. (2020). Microglia and Astrocytes in Disease: Dynamic Duo or Partners in Crime?. Trends Immunol..

[190] Vainchtein I.D., Chin G., Cho F.S., Kelley K.W., Miller J.G., Chien E.C., Liddelow S.A., Nguyen P.T., Nakao-Inoue H., Dorman L.C. (2018). Astrocyte-Derived Interleukin-33 Promotes Microglial Synapse Engulfment and Neural Circuit Development. Science.

[191] Liddelow S.A., Guttenplan K.A., Clarke L.E., Bennett F.C., Bohlen C.J., Schirmer L., Bennett M.L., Münch A.E., Chung W.S., Peterson T.C. (2017). Neurotoxic Reactive Astrocytes Are Induced by Activated Microglia. Nature.

[192] Zhang W., Xiao D., Mao Q., Xia H. (2023). Role of Neuroinflammation in Neurodegeneration Development. Signal Transduct. Target. Ther..

[193] Dunn G.A., Loftis J.M., Sullivan E.L. (2020). Neuroinflammation in Psychiatric Disorders: An Introductory Primer. Pharmacol. Biochem. Behav..

[194] Troubat R., Barone P., Leman S., Desmidt T., Cressant A., Atanasova B., Brizard B., El Hage W., Surget A., Belzung C. (2021). Neuroinflammation and Depression: A Review. Eur. J. Neurosci..

[195] Leng F., Edison P. (2021). Neuroinflammation and Microglial Activation in Alzheimer Disease: Where Do We Go from Here?. Nat. Rev. Neurol..

[196] Bradburn S., Murgatroyd C., Ray N. (2019). Neuroinflammation in Mild Cognitive Impairment and Alzheimer’s Disease: A Meta-Analysis. Ageing Res. Rev..

[197] Cai L., Yang C., Jia W., Liu Y., Xie R., Lei T., Yang Z., He X., Tong R., Gao H. (2020). Endo/Lysosome-Escapable Delivery Depot for Improving BBB Transcytosis and Neuron Targeted Therapy of Alzheimer’s Disease. Adv. Funct. Mater..

[198] Donahue J.E., Flaherty S.L., Johanson C.E., Duncan J.A., Silverberg G.D., Miller M.C., Tavares R., Yang W., Wu Q., Sabo E. (2006). RAGE, LRP-1, and Amyloid-Beta Protein in Alzheimer’s Disease. Acta Neuropathol..

[199] Yan F.L., Zheng Y., Zhao F. (2008). Di Effects of Ginkgo Biloba Extract EGb761 on Expression of RAGE and LRP-1 in Cerebral Microvascular Endothelial Cells under Chronic Hypoxia and Hypoglycemia. Acta Neuropathol..

[200] Yan F.L., Han G.L., Wu G.J. (2013). Cytotoxic Role of Advanced Glycation End-Products in PC12 Cells Treated with Β-amyloid Peptide. Mol. Med. Rep..

[201] Cho H.J., Son S.M., Jin S.M., Hong H.S., Shin D.H., Kim S.J., Huh K., Mook-Jung I. (2009). RAGE Regulates BACE1 and Abeta Generation via NFAT1 Activation in Alzheimer’s Disease Animal Model. FASEB J..

[202] Fritz G. (2011). RAGE: A Single Receptor Fits Multiple Ligands. Trends Biochem. Sci..

[203] Bai R., Guo J., Ye X.Y., Xie Y., Xie T. (2022). Oxidative Stress: The Core Pathogenesis and Mechanism of Alzheimer’s Disease. Ageing Res. Rev..

[204] Spuch C., Navarro C. (2011). Liposomes for Targeted Delivery of Active Agents against Neurodegenerative Diseases (Alzheimer’s Disease and Parkinson’s Disease). J. Drug Deliv..

[205] Noble G.T., Stefanick J.F., Ashley J.D., Kiziltepe T., Bilgicer B. (2014). Ligand-Targeted Liposome Design: Challenges and Fundamental Considerations. Trends Biotechnol..

[206] Skotland T., Hessvik N.P., Sandvig K., Llorente A. (2019). Exosomal Lipid Composition and the Role of Ether Lipids and Phosphoinositides in Exosome Biology. J. Lipid Res..

[207] Fernandes M., Lopes I., Magalhães L., Sárria M.P., Machado R., Sousa J.C., Botelho C., Teixeira J., Gomes A.C. (2021). Novel Concept of Exosome-like Liposomes for the Treatment of Alzheimer’s Disease. J. Control Release.

[208] Yang Q., Li R., Hong Y., Liu H., Jian C., Zhao S. (2024). Curcumin-Loaded Gelatin Nanoparticles Cross the Blood-Brain Barrier to Treat Ischemic Stroke by Attenuating Oxidative Stress and Neuroinflammation. Int. J. Nanomed..

[209] Cerqueira S.R., Ayad N.G., Lee J.K. (2020). Neuroinflammation Treatment via Targeted Delivery of Nanoparticles. Front. Cell Neurosci..

[210] Fournier A.P., Quenault A., De Lizarrondo S.M., Gauberti M., Defer G., Vivien D., Docagne F., MacRez R. (2017). Prediction of Disease Activity in Models of Multiple Sclerosis by Molecular Magnetic Resonance Imaging of P-Selectin. Proc. Natl. Acad. Sci. USA.

[211] Gauberti M., Fournier A.P., Docagne F., Vivien D., de Lizarrondo S.M. (2018). Molecular Magnetic Resonance Imaging of Endothelial Activation in the Central Nervous System. Theranostics.

[212] Ma J., Zhang S., Liu J., Liu F., Du F., Li M., Chen A.T., Bao Y., Suh H.W., Avery J. (2019). Targeted Drug Delivery to Stroke via Chemotactic Recruitment of Nanoparticles Coated with Membrane of Engineered Neural Stem Cells. Small.

[213] Rüster B., Göttig S., Ludwig R.J., Bistrian R., Müller S., Seifried E., Gille J., Henschler R. (2006). Mesenchymal Stem Cells Display Coordinated Rolling and Adhesion Behavior on Endothelial Cells. Blood.

[214] Sackstein R., Merzaban J.S., Cain D.W., Dagia N.M., Spencer J.A., Lin C.P., Wohlgemuth R. (2008). Ex Vivo Glycan Engineering of CD44 Programs Human Multipotent Mesenchymal Stromal Cell Trafficking to Bone. Nat. Med..

[215] Tiwari S.K., Agarwal S., Seth B., Yadav A., Nair S., Bhatnagar P., Karmakar M., Kumari M., Chauhan L.K.S., Patel D.K. (2014). Curcumin-Loaded Nanoparticles Potently Induce Adult Neurogenesis and Reverse Cognitive Deficits in Alzheimer’s Disease Model via Canonical Wnt/β-Catenin Pathway. ACS Nano.

[216] Asgarpour K., Shojaei Z., Amiri F., Ai J., Mahjoubin-Tehran M., Ghasemi F., Arefnezhad R., Hamblin M.R., Mirzaei H. (2020). Exosomal MicroRNAs Derived from Mesenchymal Stem Cells: Cell-to-Cell Messages. Cell Commun. Signal..

[217] Peshkova M., Korneev A., Suleimanov S., Vlasova I.I., Svistunov A., Kosheleva N., Timashev P. (2023). MSCs’ Conditioned Media Cytokine and Growth Factor Profiles and Their Impact on Macrophage Polarization. Stem Cell Res. Ther..

[218] Rahimi Darehbagh R., Seyedoshohadaei S.A., Ramezani R., Rezaei N. (2024). Stem Cell Therapies for Neurological Disorders: Current Progress, Challenges, and Future Perspectives. Eur. J. Med. Res..

[219] Xiao B., Hui Ng H., Takahashi R., Tan E.K. (2016). Induced Pluripotent Stem Cells in Parkinson’s Disease: Scientific and Clinical Challenges. J. Neurol. Neurosurg. Psychiatry.

[220] Li M., Chen H., Zhu M. (2022). Mesenchymal Stem Cells for Regenerative Medicine in Central Nervous System. Front. Neurosci..

[221] Gao T., Huang F., Wang W., Xie Y., Wang B. (2022). Interleukin-10 Genetically Modified Clinical-Grade Mesenchymal Stromal Cells Markedly Reinforced Functional Recovery after Spinal Cord Injury via Directing Alternative Activation of Macrophages. Cell Mol. Biol. Lett..

[222] Sajjad U., Ahmed M., Iqbal M.Z., Riaz M., Mustafa M., Biedermann T., Klar A.S. (2024). Exploring Mesenchymal Stem Cells Homing Mechanisms and Improvement Strategies. Stem Cells Transl. Med..

[223] Ghaffari-Nazari H. (2018). The Known Molecules Involved in MSC Homing and Migration. J. Stem Cell Res. Med..

[224] Bajdak-Rusinek K., Fus-Kujawa A., Buszman P., Matsuzaka Y., Yashiro R. (2024). Current Strategies and Therapeutic Applications of Mesenchymal Stem Cell-Based Drug Delivery. Pharmaceuticals.

[225] Krueger T.E.G., Thorek D.L.J., Denmeade S.R., Isaacs J.T., Brennen W.N. (2018). Concise Review: Mesenchymal Stem Cell-Based Drug Delivery: The Good, the Bad, the Ugly, and the Promise. Stem Cells Transl. Med..

[226] Maldonado V.V., Patel N.H., Smith E.E., Barnes C.L., Gustafson M.P., Rao R.R., Samsonraj R.M. (2023). Clinical Utility of Mesenchymal Stem/Stromal Cells in Regenerative Medicine and Cellular Therapy. J. Biol. Eng..

[227] Ling T.S., Chandrasegaran S., Xuan L.Z., Suan T.L., Elaine E., Nathan D.V., Chai Y.H., Gunasekaran B., Salvamani S. (2021). The Potential Benefits of Nanotechnology in Treating Alzheimer’s Disease. Biomed. Res. Int..

[228] España-Sánchez B.L., Cruz-Soto M.E., Elizalde-Peña E.A., Sabasflores-Benítez S., Roca-Aranda A., Esquivel-Escalante K., Luna-Bárcenas G. (2018). Trends in Tissue Regeneration: Bio-Nanomaterials. Tissue Regen..

[229] Nucci L.P., Silva H.R., Giampaoli V., Mamani J.B., Nucci M.P., Gamarra L.F. (2015). Stem Cells Labeled with Superparamagnetic Iron Oxide Nanoparticles in a Preclinical Model of Cerebral Ischemia: A Systematic Review with Meta-Analysis. Stem Cell Res. Ther..

[230] Ferreira R., Fonseca M.C., Santos T., Sargento-Freitas J., Tjeng R., Paiva F., Castelo-Branco M., Ferreira L.S., Bernardino L. (2016). Retinoic Acid-Loaded Polymeric Nanoparticles Enhance Vascular Regulation of Neural Stem Cell Survival and Differentiation after Ischaemia. Nanoscale.

[231] Huang D.M., Hsiao J.K., Chen Y.C., Chien L.Y., Yao M., Chen Y.K., Ko B.S., Hsu S.C., Tai L.A., Cheng H.Y. (2009). The Promotion of Human Mesenchymal Stem Cell Proliferation by Superparamagnetic Iron Oxide Nanoparticles. Biomaterials.

[232] Green J.J., Zhou B.Y., Mitalipova M.M., Beard C., Langer R., Jaenisch R., Anderson D.G. (2008). Nanoparticles for Gene Transfer to Human Embryonic Stem Cell Colonies. Nano Lett..

[233] Sun Y., Kong J., Ge X., Mao M., Yu H., Wang Y. (2023). An Antisense Oligonucleotide-Loaded Blood-Brain Barrier Penetrable Nanoparticle Mediating Recruitment of Endogenous Neural Stem Cells for the Treatment of Parkinson’s Disease. ACS Nano.

[234] Zhou X., Yuan L., Wu C., Chen C., Luo G., Deng J., Mao Z. (2018). Recent Review of the Effect of Nanomaterials on Stem Cells. RSC Adv..

[235] Chen G.Y., Pang D.W.P., Hwang S.M., Tuan H.Y., Hu Y.C. (2012). A Graphene-Based Platform for Induced Pluripotent Stem Cells Culture and Differentiation. Biomaterials.

[236] López-Ornelas A., Escobedo-Avila I., Ramírez-García G., Lara-Rodarte R., Meléndez-Ramírez C., Urrieta-Chávez B., Barrios-García T., Cáceres-Chávez V.A., Flores-Ponce X., Carmona F. (2023). Human Embryonic Stem Cell-Derived Immature Midbrain Dopaminergic Neurons Transplanted in Parkinsonian Monkeys. Cells.

[237] Hachimi-Idrissi S., Fischer S. (2023). Stem Cell Therapy in Neurological Disorders: Promises and Concerns. Open Explor..

[238] Willerth S.M. (2011). Neural Tissue Engineering Using Embryonic and Induced Pluripotent Stem Cells. Stem Cell Res. Ther..

[239] Huang Y., Wu Q., Tam P.K.H. (2022). Immunomodulatory Mechanisms of Mesenchymal Stem Cells and Their Potential Clinical Applications. Int. J. Mol. Sci..

[240] Yan J., Huang L., Feng J., Yang X. (2023). The Recent Applications of PLGA-Based Nanostructures for Ischemic Stroke. Pharmaceutics.

[241] Wang X., Wang Q., Xia Z., Yang Y., Dai X., Zhang C., Wang J., Xu Y. (2024). Mesenchymal Stromal Cell Therapies for Traumatic Neurological Injuries. J. Transl. Med..

[242] Hasan A., Deeb G., Rahal R., Atwi K., Mondello S., Marei H.E., Gali A., Sleiman E. (2017). Mesenchymal Stem Cells in the Treatment of Traumatic Brain Injury. Front. Neurol..

[243] Chen Q.H., Liu A.R., Qiu H.B., Yang Y. (2015). Interaction between Mesenchymal Stem Cells and Endothelial Cells Restores Endothelial Permeability via Paracrine Hepatocyte Growth Factor in Vitro. Stem Cell Res. Ther..

[244] Barry M., Trivedi A., Miyazawa B., Vivona L.R., Shimmin D., Pathipati P., Keane C., Cuschieri J., Pati S. (2024). Regulation of Vascular Endothelial Integrity by Mesenchymal Stem Cell Extracellular Vesicles after Hemorrhagic Shock and Trauma. J. Transl. Med..

[245] Hey S., Linder S. (2024). Matrix Metalloproteinases at a Glance. J. Cell Sci..

[246] Cunningham M.C., Bolay H., Scouten C.W., Moore C., Jacoby D., Moskowitz M., Sorensen J.C., Kelly P.J., Breeze R.E., Bruce J.N. (2004). Preclinical Evaluation of a Novel Intracerebral Microinjection Instrument Permitting Electrophysiologically Guided Delivery of Therapeutics. Neurosurgery.

[247] Saleh R.O., Majeed A.A., Margiana R., Alkadir O.K.A., Almalki S.G., Ghildiyal P., Samusenkov V., Jabber N.K., Mustafa Y.F., Elawady A. (2024). Therapeutic Gene Delivery by Mesenchymal Stem Cell for Brain Ischemia Damage: Focus on Molecular Mechanisms in Ischemic Stroke. Cell Biochem. Funct..

[248] Eggenhofer E., Benseler V., Kroemer A., Popp F.C., Geissler E.K., Schlitt H.J., Baan C.C., Dahlke M.H., Hoogduijn M.J. (2012). Mesenchymal Stem Cells Are Short-Lived and Do Not Migrate beyond the Lungs after Intravenous Infusion. Front. Immunol..

[249] Fischer U.M., Harting M.T., Jimenez F., Monzon-Posadas W.O., Xue H., Savitz S.I., Laine G.A., Cox C.S. (2009). Pulmonary Passage Is a Major Obstacle for Intravenous Stem Cell Delivery: The Pulmonary First-Pass Effect. Stem Cells Dev..

[250] Sharma A., Sane H., Gokulchandran N., Badhe P., Kulkarni P., Pai S., Varghese R., Paranjape A. (2017). Stem Cell Therapy in Pediatric Neurological Disabilities. Phys. Disabil. Ther. Implic..

[251] Jiang Y., Pan X., Yu T., Wang H. (2023). Intranasal Administration Nanosystems for Brain-Targeted Drug Delivery. Nano Res..

[252] Zhang Y.T., He K.J., Zhang J.B., Ma Q.H., Wang F., Liu C.F. (2021). Advances in Intranasal Application of Stem Cells in the Treatment of Central Nervous System Diseases. Stem Cell Res. Ther..

[253] Chen T., Dai Y., Hu C., Lin Z., Wang S., Yang J., Zeng L., Li S., Li W. (2024). Cellular and Molecular Mechanisms of the Blood–Brain Barrier Dysfunction in Neurodegenerative Diseases. Fluids Barriers CNS.

[254] Tang X., Deng P., Li L., He Y., Wang J., Hao D., Yang H. (2024). Advances in Genetically Modified Neural Stem Cell Therapy for Central Nervous System Injury and Neurological Diseases. Stem Cell Res. Ther..

[255] Yari H., Mikhailova M.V., Mardasi M., Jafarzadehgharehziaaddin M., Shahrokh S., Thangavelu L., Ahmadi H., Shomali N., Yaghoubi Y., Zamani M. (2022). Emerging Role of Mesenchymal Stromal Cells (MSCs)-Derived Exosome in Neurodegeneration-Associated Conditions: A Groundbreaking Cell-Free Approach. Stem Cell Res. Ther..

[256] Ghareghani M., Arneaud A., Rivest S. (2024). The Evolution of Mesenchymal Stem Cell-Derived Neural Progenitor Therapy for Multiple Sclerosis: From Concept to Clinic. Front. Cell Neurosci..

[257] Li X., Sundström E. (2022). Stem Cell Therapies for Central Nervous System Trauma: The 4 Ws—What, When, Where, and Why. Stem Cells Transl. Med..

[258] Palma-Tortosa S., Coll-San Martin B., Kokaia Z., Tornero D. (2021). Neuronal Replacement in Stem Cell Therapy for Stroke: Filling the Gap. Front. Cell Dev. Biol..

[259] Tornero D. (2022). Neuronal Circuitry Reconstruction after Stem Cell Therapy in Damaged Brain. Neural Regen. Res..

[260] Dekmak A.S., Mantash S., Shaito A., Toutonji A., Ramadan N., Ghazale H., Kassem N., Darwish H., Zibara K. (2018). Stem Cells and Combination Therapy for the Treatment of Traumatic Brain Injury. Behav. Brain Res..

[261] Cox C.S., Baumgartner J.E., Harting M.T., Worth L.L., Walker P.A., Shah S.K., Ewing-Cobbs L., Hasan K.M., Day M.C., Lee D. (2011). Autologous Bone Marrow Mononuclear Cell Therapy for Severe Traumatic Brain Injury in Children. Neurosurgery.

[262] Kumar A., Dudhal S., Abinaya Sundari T., Sunkara M., Usman H., Varshney A., Mukhopadhyay A. (2016). Dopaminergic-Primed Fetal Liver Mesenchymal Stromal-like Cells Can Reverse Parkinsonian Symptoms in 6-Hydroxydopamine-Lesioned Mice. Cytotherapy.

[263] Venkataramana N.K., Kumar S.K.V., Balaraju S., Radhakrishnan R.C., Bansal A., Dixit A., Rao D.K., Das M., Jan M., Gupta P.K. (2010). Open-Labeled Study of Unilateral Autologous Bone-Marrow-Derived Mesenchymal Stem Cell Transplantation in Parkinson’s Disease. Transl. Res..

[264] Kim Y.J., Park H.J., Lee G., Bang O.Y., Ahn Y.H., Joe E., Kim H.O., Lee P.H. (2009). Neuroprotective Effects of Human Mesenchymal Stem Cells on Dopaminergic Neurons through Anti-Inflammatory Action. Glia.

[265] Park H.J., Shin J.Y., Kim H.N., Oh S.H., Song S.K., Lee P.H. (2015). Mesenchymal Stem Cells Stabilize the Blood-Brain Barrier through Regulation of Astrocytes. Stem Cell Res. Ther..

[266] Shin J.Y., Park H.J., Kim H.N., Oh S.H., Bae J.S., Ha H.J., Lee P.H. (2014). Mesenchymal Stem Cells Enhance Autophagy and Increase β-Amyloid Clearance in Alzheimer Disease Models. Autophagy.

[267] Lee J.K., Jin H.K., Bae J. (2009). sung Bone Marrow-Derived Mesenchymal Stem Cells Reduce Brain Amyloid-β Deposition and Accelerate the Activation of Microglia in an Acutely Induced Alzheimer’s Disease Mouse Model. Neurosci. Lett..

[268] Kim H.J., Seo S.W., Chang J.W., Lee J.I., Kim C.H., Chin J., Choi S.J., Kwon H., Yun H.J., Lee J.M. (2015). Stereotactic Brain Injection of Human Umbilical Cord Blood Mesenchymal Stem Cells in Patients with Alzheimer’s Disease Dementia: A Phase 1 Clinical Trial. Alzheimers Dement..

[269] Krakora D., Mulcrone P., Meyer M., Lewis C., Bernau K., Gowing G., Zimprich C., Aebischer P., Svendsen C.N., Suzuki M. (2013). Synergistic Effects of GDNF and VEGF on Lifespan and Disease Progression in a Familial ALS Rat Model. Mol. Ther..

[270] Karussis D., Karageorgiou C., Vaknin-Dembinsky A., Gowda-Kurkalli B., Gomori J.M., Kassis I., Bulte J.W.M., Petrou P., Ben-Hur T., Abramsky O. (2010). Safety and Immunological Effects of Mesenchymal Stem Cell Transplantation in Patients with Multiple Sclerosis and Amyotrophic Lateral Sclerosis. Arch. Neurol..

[271] Hanson L.R., Frey W.H. (2008). Intranasal Delivery Bypasses the Blood-Brain Barrier to Target Therapeutic Agents to the Central Nervous System and Treat Neurodegenerative Disease. BMC Neurosci..

[272] Salama M., Sobh M., Emam M., Abdalla A., Sabry D., El-Gamal M., Lotfy A., El-Husseiny M., Sobh M., Shalash A. (2017). Effect of Intranasal Stem Cell Administration on the Nigrostriatal System in a Mouse Model of Parkinson’s Disease. Exp. Ther. Med..

[273] Lim J.C., Wolpaw A.J., Caldwell M.A., Hladky S.B., Barrand M.A. (2007). Neural Precursor Cell Influences on Blood-Brain Barrier Characteristics in Rat Brain Endothelial Cells. Brain Res..

[274] Shen Q., Goderie S.K., Jin L., Karanth N., Sun Y., Abramova N., Vincent P., Pumiglia K., Temple S. (2004). Endothelial Cells Stimulate Self-Renewal and Expand Neurogenesis of Neural Stem Cells. Science.

[275] Stenman J.M., Rajagopal J., Carroll T.J., Ishibashi M., McMahon J., McMahon A.P. (2008). Canonical Wnt Signaling Regulates Organ-Specific Assembly and Differentiation of CNS Vasculature. Science.

[276] Daneman R., Agalliu D., Zhou L., Kuhnert F., Kuo C.J., Barres B.A. (2009). Wnt/Beta-Catenin Signaling Is Required for CNS, but Not Non-CNS, Angiogenesis. Proc. Natl. Acad. Sci. USA.

[277] Lippmann E.S., Azarin S.M., Kay J.E., Nessler R.A., Wilson H.K., Al-Ahmad A., Palecek S.P., Shusta E.V. (2012). Derivation of Blood-Brain Barrier Endothelial Cells from Human Pluripotent Stem Cells. Nat. Biotechnol..

[278] Armulik A., Genové G., Mäe M., Nisancioglu M.H., Wallgard E., Niaudet C., He L., Norlin J., Lindblom P., Strittmatter K. (2010). Pericytes Regulate the Blood–Brain Barrier. Nature.

[279] Dauchy S., Miller F., Couraud P.O., Weaver R.J., Weksler B., Romero I.A., Scherrmann J.M., De Waziers I., Declèves X. (2009). Expression and Transcriptional Regulation of ABC Transporters and Cytochromes P450 in HCMEC/D3 Human Cerebral Microvascular Endothelial Cells. Biochem. Pharmacol..

[280] Lyck R., Ruderisch N., Moll A.G., Steiner O., Cohen C.D., Engelhardt B., Makrides V., Verrey F. (2009). Culture-Induced Changes in Blood-Brain Barrier Transcriptome: Implications for Amino-Acid Transporters in Vivo. J. Cereb. Blood Flow. Metab..

[281] Butt A.M., Jones H.C., Abbott N.J. (1990). Electrical Resistance across the Blood-Brain Barrier in Anaesthetized Rats: A Developmental Study. J. Physiol..

[282] Bell R.D., Winkler E.A., Singh I., Sagare A.P., Deane R., Wu Z., Holtzman D.M., Betsholtz C., Armulik A., Sallstrom J. (2012). Apolipoprotein E Controls Cerebrovascular Integrity via Cyclophilin A. Nature.

[283] Vasudevan A., Long J.E., Crandall J.E., Rubenstein J.L.R., Bhide P.G. (2008). Compartment-Specific Transcription Factors Orchestrate Angiogenesis Gradients in the Embryonic Brain. Nat. Neurosci..

[284] Israel M.A., Yuan S.H., Bardy C., Reyna S.M., Mu Y., Herrera C., Hefferan M.P., Van Gorp S., Nazor K.L., Boscolo F.S. (2012). Probing Sporadic and Familial Alzheimer’s Disease Using Induced Pluripotent Stem Cells. Nature.

[285] Hernando S., Nikolakopoulou P., Voulgaris D., Hernandez R.M., Igartua M., Herland A. (2022). Dual Effect of TAT Functionalized DHAH Lipid Nanoparticles with Neurotrophic Factors in Human BBB and Microglia Cultures. Fluids Barriers CNS.

